# Cell-Based Antioxidant Properties and Synergistic Effects of Natural Plant and Algal Extracts Pre and Post Intestinal Barrier Transport

**DOI:** 10.3390/antiox11030565

**Published:** 2022-03-16

**Authors:** Christophe Furger, Camille Gironde, Mylène Rigal, Cécile Dufour, Damien Guillemet

**Affiliations:** 1 Anti Oxidant Power—AOP/LAAS/CNRS, 7 Avenue du Colonel Roche, 31400 Toulouse, France; cgironde@laas.fr (C.G.); mrigal@laas.fr (M.R.); cdufour@antioxidant-power.com (C.D.); 2 Nexira, 129 Chemin de Croisset, 76000 Rouen, France

**Keywords:** antioxidant, synergies, AOP1, Nrf2, epithelium passage, grape, clove, immortelle, hamamelis

## Abstract

In this work, both direct and indirect cell-based antioxidant profiles were established for 27 plant extracts and 1 algal extract. To evaluate the direct antioxidant effects, fluorescent AOP1 cell assay was utilized, which measures the ability of different samples to neutralize intracellular free radicals produced by a cell-based photo-induction process. As the intestinal barrier is the first cell line crossed by the product, dose response curves obtained from Caco-2 cells were used to establish EC_50_ values for 26 out of the 28 natural extracts. Among them, 11 extracts from *Vitis*, *Hamamelis*, *Syzygium*, *Helichrysum*, *Ilex* and *Ribes* genera showed remarkable EC_50_s in the range of 10 µg/mL. In addition to this, a synergistic effect was found when combinations of the most potent extracts (*S. aromaticum, H. italicum, H. virginiana, V. vinifera*) were utilized compared to extracts alone. Indirect antioxidant activities (i.e., the ability of cells to trigger antioxidant defenses) were studied using the ARE/Nrf2 luminescence reporter-gene assay in HepG2 cells, as liver is the first organ crossed by an edible ingredient once it enters in the bloodstream. Twelve extracts were subjected to an intestinal epithelial barrier passage in order to partially mimic intestinal absorption and show whether basolateral compartments could maintain direct or indirect antioxidant properties. Using postepithelial barrier samples and HepG2 cells as a target model, we demonstrate that indirect antioxidant activities are maintained for three extracts, *S. aromaticum*, *H. virginiana* and *H. italicum*. Our experimental work also confirms the synergistic effects of combinations of post-intestinal barrier compartments issued from apical treatment with these three extracts. By combining cell-based assays together with an intestinal absorption process, this study demonstrates the power of cell systems to address the issue of antioxidant effects in humans.

## 1. Introduction

In order to maintain their homeostasis, plants and algae are well-known to deploy rapid and efficient antioxidant strategies against drastic environmental challenges [1]. Carotenoids (with more than 700 identified species) [2] and flavonoids (with 7 main families and more than 10,000 identified species) [3,4], together with other classical nonenzymatic antioxidants, such as glutathione, ascorbate and α-tocopherol, comprise the main breeding ground of compounds than can be extracted from plants for their antioxidant properties [5].

The detailed study of different antioxidant properties in plants or plant extracts has been limited for a long time, as chemical tools such as HAT (hydrogen atop transfer) and SET (single-electron transfer) approaches [6] provide information in the form of an antioxidant capacity (AC) only. They give no clues about the actual antioxidant effect expected in living organisms [7].

Apart from animal studies, various cell and assay models have been developed in the last decade to overcome this issue [8,9]. The HepG2 cell line, which originates from the human liver, remains the most common human cell model used for antioxidant analysis, especially when working on food supplements, as liver is the first target organ following the entry of nutriments in the gastrointestinal tract [10].

The most recognized cell-based assay available to date for antioxidant activity measurement uses the so-called Keap1/Nrf2/ARE stress-response pathway, which is also called the “master regulator of stress response”. This canonical pathway regulates the transcription of a multitude of proteins involved in cellular antioxidant systems, such as the redoxin system, the OH-1 antioxidant and NQO1 cytoprotective enzymes [11]. They are all involved in the cell’s defense strategy against oxidative attacks. Stable ARE-driven firefly luciferase reporter cell lines now provide a useful tool to follow antioxidant effects at the cell level [12]. Different studies have shown that numerous phytochemicals act by activating the Keap1/Nrf2/ARE pathway [13,14,15,16]. This assay was also applied successfully to screen plant extracts according to the level of their Antioxidant Response Element (ARE) transcriptional activation. In a study carried out in HepG2 cells, 28 out of 45 (62.2%) phytochemicals and plant extracts, including coffee, broccoli and spices showed significant ARE activities [17]. More ambitiously, a high throughput screening campaign counting no less than 280 methanolic extracts from more than 181 plants collected in Panama was conducted using an Nrf2/ARE assay performed on ARE/luciferase stably transfected AREc32 cells [18]. The assay proved to be robust enough to be conducted in 384-microwell plates, and a significant increase in the ARE transcription machinery was observed (57.5%) for 161 extracts. This methodology appeared to be the most effective and discriminative, since the in vitro antioxidant capacity (AC) evaluated in parallel was positive without any useful information for 93.5% of the 280 extracts.

Tackling antioxidant effects by looking at gene expression partially answers the question as to whether direct free-radical-quenching processes can also be involved at the cell level or not. These processes are much harder to detect and assess on cell systems due to the very small half-lives of free radicals, in the order of nano- to milli-seconds. In addition to this, their production can also be difficult to control. Recent developments in cell biology and energy transfer technologies led to the AOP1 live-cell assay, which allows the monitoring of free-radical production by a photoinduction process involving an asymmetrical cyanine fluorescent biosensor located in the intracellular compartment [19]. As for the Nrf2/ARE assay, the AOP1 live-cell assay facilitates dose–response studies and has already been successful in classifying many plant extracts and standard antioxidant quenchers. These studies were undertaken based on the intracellular free-radical-neutralizing properties in HepG2 and other cell models [20]. The current list of botanical extracts was determined through bibliographical review and the European regulatory interpretations that allow their use as antioxidant botanical extracts for intended oral use.

The present work represents the first attempt to screen plant extracts on both direct (intracellular free-radical-quenching by AOP1) and indirect (gene expression by Nrf2/ARE reporter-gene system) antioxidant cell-based assays. In order to get a full picture of antioxidant effects in relation to the human organism, extracts showing the most interesting profiles were then submitted to a Transwell cellular intestinal barrier transport model in order to analyze the remaining antioxidant effects present in the basolateral postbarrier function compartments. Combinations of some extracts were eventually assayed with the hope of finding synergistic effects.

## 2. Material and Methods

### 2.1. Materials and Reagents

Thiazole orange (TO) and sulforaphane were purchased from Sigma-Aldrich (Saint-Quentin Fallavier, France). Gibco DMEM (high glucose, GlutaMAX supplement and pyruvate), fetal bovine serum (FBS) (HyClone, Logan, UT, USA), pen-strep solution (100X) (Gibco), 0.05% Trypsin-EDTA (HyClone), Gibco DPBS without Calcium, Magnesium (1X), Gibco Selective Antibiotic Geneticin (G418) (50 mg/mL) and Transwell (3402, Corning, NY, USA) were purchased from Thermo Fisher Scientific (Illkirch-Graffenstaden, France). A ONE-Step Luciferase Assay System was purchased from BPS Bioscience (catalogue number 60690, San Diego, CA, USA). HepG2 and Caco-2 cell lines were respectively purchased from the ATCC (catalog number HB8065, LGC Standards, Molsheim, France) and given by Led Engineering Development (LED, Montauban, France). A HepG2 ARE reporter cell line (Nrf2 antioxidant pathway) was purchased from BPS Bioscience (catalogue number 60513).

### 2.2. Sample Extraction Process

All plant extracts were sourced by Nexira. The list of botanical extracts is presented in Table 1, where scientific botanical names and the plant parts used are presented. The types of extraction process are also reported. Basically, the first-stage extraction used water as a solvent. S/L aqueous extract designates Solid/Liquid extraction, where the botanical raw material under dried form was extracted in an aqueous phase and separated by centrifugation. For a few specific botanical extracts, a purification stage was added to the first-stage extraction, notably for grape, as this source has been widely explored in terms of antioxidant properties, and its activities could differ because of the extraction process. All the extracts were dried and used in powder form, except for AI, AJ, AP and BB, which were used in concentrated liquid, as they were not dryable. All data here are reported in a dried-form equivalency unit of the native extract.

### 2.3. Sample Preparation

Stock solutions of compounds and samples were prepared in advance. Apart from the samples already in liquid format, all samples were solubilized in a DMEM culture medium. The solutions were centrifuged at 8700 rpm for 10 min, and supernatant was collected, aliquoted and stored at −20 °C. For the dose–response experiments, different concentrations were obtained by serial factor-2 dilutions. The experiments were carried out in 96-well microplates using the 60 most-centered wells. The samples were incubated in a serum-free medium in order to avoid potential interaction with components of the serum. Each experiment was performed in triplicates (AOP1) or duplicates (ARE/Nrf2), including the negative control (culture medium, ethanol or DMSO), according to the solvent used. Apart from the last series of data presented in Section 3.5, all conditions were subjected to two independent sets of experiments.

### 2.4. Cell Culture

The HepG2 cells (passages 15–35) were cultured at 37 °C under 5% CO_2_ in a complemented (FBS 10%, pen-strep 1X) GlutaMAX DMEM medium. For the HepG2/Nrf2 cells (passages 4–16), the cells were cultured at 37 °C/5% CO_2_ in a GlutaMAX DMEM medium complemented with a 10% FBS, 1X pen-strep solution and 50 mg/mL G418. The Caco-2 cells (passage 20 to 35) were grown in a GlutaMAX DMEM medium complemented with 20% FBS and 1X pen-strep. The cells were grown up to 70–80% confluence before transfer (10^6^ cells/mL for HepG2 and 4 × 10^5^ cells/mL for Caco-2) to clear-bottom 96-well microplates.

### 2.5. AOP1 Assay Experimental Protocol

The cells were first incubated for 4 h at 37 °C in 5% CO_2_ with each experimental condition. TO was added to the cells (4 μM, 1 h, 37 °C in 5% CO_2_). Fluorescence emission (expressed as RFU, Relative Fluorescence Unit) was measured (flash number 0) using a Varioskan Flash Spectral Scanning Multimode Reader (Thermo Fisher Scientific, Waltham, MA, USA) set up at 505/535 (excitation/emission) nm. The microplates were illuminated (470 nm, 24 mJ/cm^2^) using a light application device (24 LEDs, each centered on the intersection of a square of 4 wells) provided by LED (Montauban, France). Fluorescence emission was measured again immediately after illumination (flash number 1) before applying the light again. This cycle was repeated (flash number 2, 3, 4, etc.) until the fluorescence emission reached a plateau.

Kinetic profiles were normalized using Prism8 (GraphPad, San Diego, CA, USA), according to control data and expressed as a cellular Antioxidant Index (AI) according to the formula:

AOP index (%) = 100 − 100 (_0_∫^20^ RFU_sample_/_0_∫^20^ RFU_control_)

Dose–response curves, obtained by compiling AIs according to a logarithm (10) of the sample concentration, were submitted to a sigmoid fit according to the formula:

AOP index = AOP index_min_ + (AOP index_max_ − AOP index_min_)/(1 + 10^(Log(EC^_50_^-SC)^
^∗ HS)^)

where SC = sample concentration and HS = hillslope = slope coefficient of the tangent at the inflection point. EC_50_ (50% efficacy concentration), EC_10_ and EC_90_ are then evaluated according to the fit. *p* values were produced when necessary, using a two-tailed, unpaired *t*-test.

### 2.6. ARE/Nrf2 Reporter-Gene System

The HepG2/Nrf2 cells were treated with conditions for 17 h; then, they were treated with a mix (BPS Bioscience, USA) comprising cell lysis solution and luciferin (substrate of luciferase) for 40 min. Luminescence was read on a Varioskan Flash Spectral Scanning Multimode Reader. Relative Luminescence values (RLU) reveal luciferase gene expression following ARE promotion. The results are presented as Fold Increase (FI) in the control values at t = 20 min according to the formula:

Fold Increase (FI) = RLUsample/RLUcontrol

As above, dose–response curves were submitted to a sigmoid fit, and EC_50_ (50% efficacy concentration) was evaluated according to the fit. *p* values were produced when necessary, using a two-tailed, unpaired *t*-test.

### 2.7. Transwell Intestinal Epithelial Barrier Model

The Caco-2 cells (passages 20–35) were cultured at 37 °C in 5% CO_2_ in a complemented (FBS 20%, pen-strep 1X) GlutaMAX DMEM medium. The cells were allowed to grow to 70–80% confluence. The basolateral compartments of the Transwell polycarbonate membrane cell culture inserts (12-well microplate, Corning 3402) were filled with 1.5 mL of the medium. Next, 500 µL of cells (760,000 cells) were added in the apical compartments, and the cells were grown for 21 days at 37 °C/5% CO_2_. The culture medium (500 µL) was replaced in the apical compartments every other day. After 21 days, the medium in the apical compartments was replaced by the samples, all at 10 mg/mL concentration, apart from *H. italicum* at 1 mg/mL, for 4 h at 37 °C/5% CO_2_. At the end of the incubation, both compartments were collected for further experiments. Transepithelial Electrical Resistance (TEER) was measured before sample addition, and again after 4 h incubation, with an ohmmeter (MERS00002 Millipore Voltmètre-Ohmmètre Millicell-ER).

## 3. Results

### 3.1. Screening Campaign Based on Extract Antioxidant Properties

**Figure 1:**
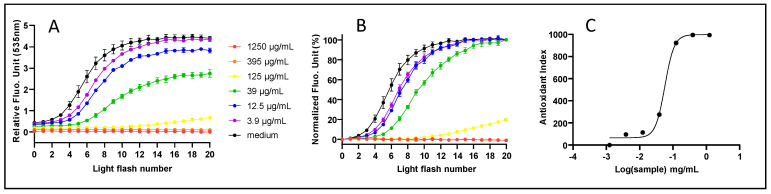
AOP1 direct antioxidant assay. The example of Caco-2 cells treated for 4 h with *Syzygium aromaticum* extract on a dose–response mode. All other extracts were subjected to the same protocol and data analyses. (**A**) Kinetic raw data showing the effect of light flashes on fluorescence level; (**B**) same data normalized to T = 0 values; (**C**) Antioxidant Index (AI)-based dose–response and sigmoid fit curve used for Efficacy Concentration (EC) estimations.

All 28 samples (Table 1), named AA to BF for blind testing, were assayed on a dose–response mode using AOP1 technology. AOP1 measures the ability of the samples to neutralize intracellular free radicals and other ROS produced by photoinduction under light flashes at 480 nm. In untreated cells, ROS production results in an increase in the fluorescence level that occurs after recurrent light applications (Figure 1A, black curve). Any condition neutralizing the ROS level inside the cell will delay or even abolish the fluorescence level modulation observed in untreated cells. For example, Figure 1 depicts the case of Caco-2 cells in culture, treated for 4 h with increasing concentrations of *S. aromaticum* extract. At the highest concentrations (>125 µg/mL), a full effect (flat curve) is observed. Inversely, a control-like profile is observed at the lowest concentrations (<12.5 µg/mL). Integration of the signal (AUC) after data normalization (Figure 1B) gives an Antioxidant Index (AI) for each condition, which enables the calculation of the EC_50_ after sigmoid fit in a dose–response mode. In this specific case, the EC_50_ has been established at 57.78 µg/mL, the EC_10_ at 28.29 µg/mL and the EC_90_ at 118 µg/mL (see Figure S1 for details).

**Figure 2:**
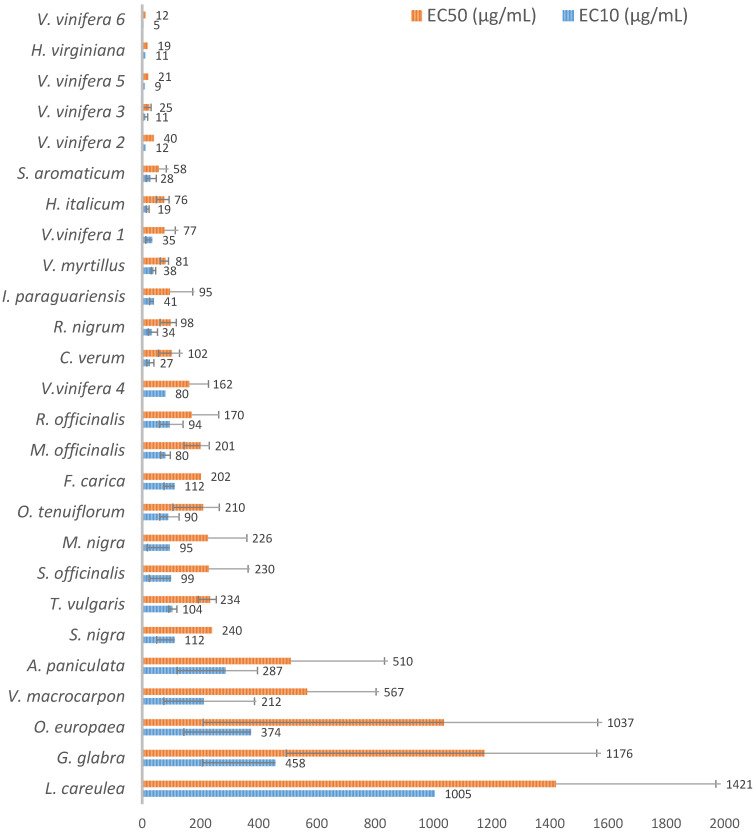
Natural extract screening campaign using AOP1 direct antioxidant assay on Caco-2 cells. EC_10_/EC_50_s could be calculated from dose–response curves for 26 out of the 28 extracts depicted in Table 1. Data is presented according to the increasing values of the EC_50_ in µg/mL. Error bars represent the 95% Confident Index on the EC (when calculable).

All samples were subjected to the same protocol. The EC_50_s and EC_10_s obtained for each extract are presented in Figure 2. Most *V. vinifera* samples, along with *H. virginiana*, *S. aromaticum*, *H. italicum*, *V. myrtillus*, *I. paraguariensis* and *R. nigrum*, gave EC_50_s in the 10 µg/mL range. Only three extracts, *O. europaea*, *G. glabra* and *L. caerulea,* gave EC_50_s above 1 mg/mL. All measured EC_10_s were ≤1 mg/mL. Only one extract did not show any direct antioxidant properties: *Undaria pinnatifida* (wakame), an alga that differs from the other samples, which are all from plant origin. One other extract, *Malpighia emarginata* (acerola, fruit juice extraction) only gave a partial effect, with a maximum Antioxidant Index (AI) = 510 (out to 1000) and with no possibility for EC_50_ evaluation. A few of the samples also showed some cytotoxic effects at the highest concentrations, as observed by fluorescence values above control value at T = 0 (Figure S1). All together, these results show that most of the tested extracts present a strong, direct antioxidant effect by neutralizing the free radical species produced by the Caco-2 cells in culture.

### 3.2. Effect of Extract Combinations on Antioxidant Properties

**Figure 3:**
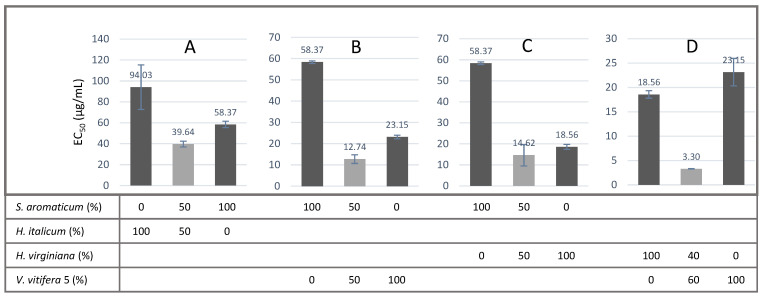
Illustration of synergistic effects observed with various extract combinations on Caco-2 cells. Only the combination with the highest variation between observed and theoretical EC_50_s is shown. Complete results are presented in Table 2. EC_50_ variation in (**A**) = −47.98%, in (**B**) = −68.74%, in (**C**) = −61.99% and in (**D**) = −84.52%. A negative percentage value indicates a synergistic effect.

Based on the results of the screening campaign, the four extracts that performed the best, namely *V. vinifera*, *H. virginiana*, *S. aromaticum* and *H. italicum,* were used in different proportions in order to highlight the potential synergistic effects of extract combinations. A synergistic effect is characterized when the measured EC_50_ of a combination is significantly lower than the theoretical EC_50_s that would be expected for the combination, knowing the EC_50_s of the 100% extracts. Eleven combinations were tested. The results are presented in Figure 3 and Table 2. The highest synergistic effect was found in *H. virginiana/V. vinifera* in a 40/60 proportion, followed by *S. aromaticum*/*V. vinifera*—50/50, *S. aromaticum/H. virginiana*—50/50 and *S. aromaticum/H. italicum*—50/50.

Minor differences in the EC_50_ values in 100% extract samples between Figure 2 and Figure 3 are due to a different mode of EC_50_ calculation. The data comes from the same set, but in Figure 2, the sigmoid fit was obtained from the mean of the triplicate RFU values, and in Figure 3, from mean of the EC_50_ values calculated from each of the replicates, the latter calculation mode being used for the evaluation of EC_50_s of the mixed extracts to remain consistent.

Twelve extracts from the screening campaign were also selected for further experiments. In this case, we considered the ones that presented the lowest EC_50_s in the Caco-2 cells, in the range of 11 to 200 µg/mL (Figure 1). This new set of extracts were further tested with the same AOP1 assay, this time using the HepG2 human liver cell model (Table 3). All samples but one (*H. italicum*, for which no full effect nor sigmoid fit were obtained) gave EC_50_s in the range of 15 to 308 µg/mL, consistent with the previous data obtained in the Caco-2 cells. The extracts gave results in the same order in both cell models, apart from *V. myrtillus*, which was three times more potent in the Caco-2 cells, and *C. verum*, which was twice as potent in the HepG2 cells.

### 3.3. Effect of Selected Extracts on the ARE/Nrf2 Antioxidant Pathway

**Figure 4:**
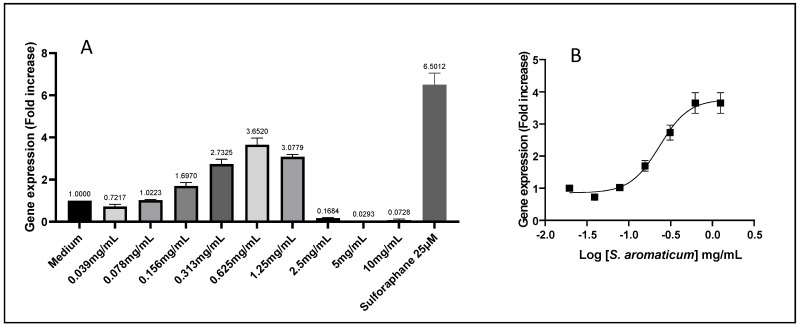
Indirect antioxidant ARE/Nrf2 assay data obtained for *Syzygium aromaticum* extract (given as an example) in HepG2/Nrf2 cells. All other extracts were subjected to the same protocol and data analyses. (**A**) Assay of increasing concentrations showing the typical bell-shaped profile (see text for explanation). Sulforaphane 25 µM was used as positive control; (**B**) dose–response and sigmoid fit curve used for Efficacy Concentration (EC) estimations.

In order to get a better idea of the antioxidant effects of the extracts, we then tested the ability of the 12 selected samples to modulate the ARE/Nrf2 transcriptional pathway using a reporter-gene system established in the HepG2 cells by stable transfection. An example of the results is given in Figure 4 for the case of the *S. aromaticum* extract. The gene expression level is given after 17 h of treatment. The dose–response profile shows a dose-dependent increase in ARE/Nrf2 gene expression (in comparison to constitutive ARE/Nrf2 gene expression given by a negative control *medium* condition) up to a maximum, followed by a strong decrease in gene expression due to cytotoxic effects at higher concentrations (here above 1.25 mg/mL), in agreement with classical dose–response ARE/Nrf2 gene expression profiles. The gene expression level of sulforaphane (25 µM) is given as a positive control. In the specific example of *S. aromaticum*, a maximum of 3.65 times of activity was reached at 625 µg/mL concentration (Figure 4A). High concentrations (2.5–10 mg/mL in the example presented in Figure 4B) for which gene expression has dropped due to cytotoxicity were discarded from the dose–response curve.

The calculated EC_50_s with corresponding determination coefficients (from duplicates) are presented in Table 4. All samples but the two *V. vinifera* extracts from seeds showed measurable EC_50_s, but in the range of 233 to 2878 µg/mL, well above those obtained with the AOP1 direct antioxidant assay.

### 3.4. Caco-2 Intestinal Transport Study and Antioxidant Properties of Post-Intestinal Barrier Samples

The intestinal transepithelial passage study was performed on a Transwell microplate system after 21 days of cell culture. The integrity of the Caco-2 monolayer (intercellular space closed by tight junctions) was evaluated by measuring the transepithelial electrical resistance (TEER) before and at the end of the 4 h treatment with extract samples. The results showed that the TEER values, initially in the range of 538 to 847 Ω × cm^2^, were still significantly high after treatment, in the range of 398 to 775 Ω (446 to 839 Ω × cm^2^), with a loss in the range of 3.90 to 37.91% between the two conditions. The comparison of these data is summarized in Table 5.

**Figure 5:**
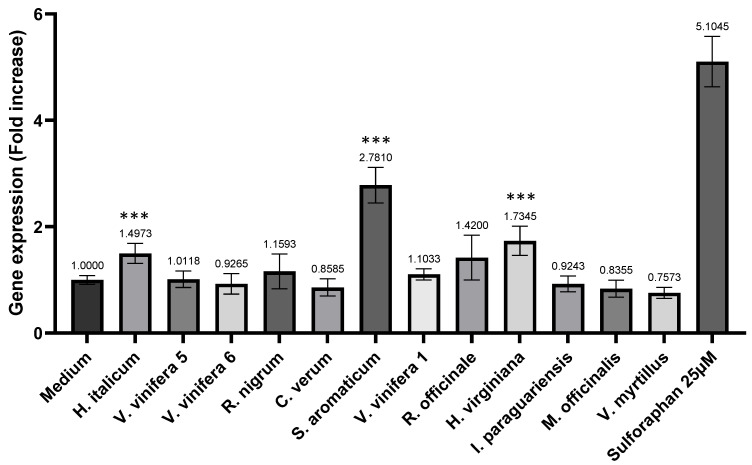
Effect of post-intestinal barrier transport compartments on ARE/NRF2 pathway activation in HepG2 cells. Results are expressed as fold increase compared to constitutive medium ARE/Nrf2 gene expression. Values represent means of quadruplicates obtained from two independent intestinal barrier transport experiments. Sulforaphane 25 µM data is provided as positive control; *** *p* < 0.005.

Each of the apical compartments of the Transwell wells were loaded with one of the 12 selected extract samples. After 4 h of incubation, the basolateral compartments were collected for further experiments. An AOP1 assay was applied to the HepG2 cells for all basolateral samples, but none of them showed any effect (Figure S2). These results imply that the direct antioxidant activity was not transferred to the Caco-2 cell basolateral compartments at a measurable level. However, some positive results were found when the same samples were then subjected to the ARE/Nrf2 assay. The three basolateral samples from *S. aromaticum*, *H. italicum* and *H. virginiana* showed a significant increase in ARE/Nrf2 gene expression, with *S. aromaticum* reaching 2.78 times the constitutive level presented by the negative control (culture medium) (Figure 5).

### 3.5. ARE/Nrf2 Antioxidant Properties of Post-Intestinal Barrier Combinations

**Figure 6:**
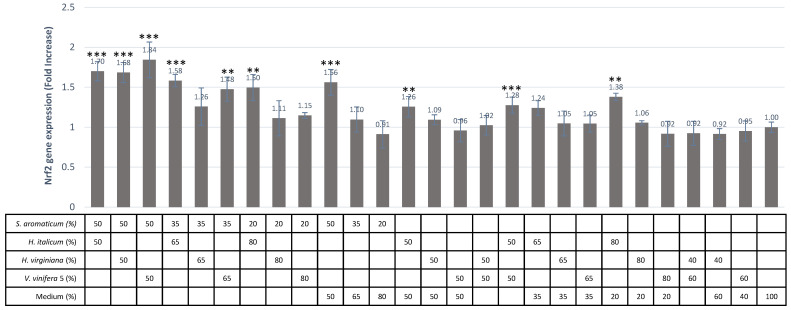
Effect of combinations of post-intestinal barrier compartments on ARE/NRF2 pathway in HepG2 cells. Results are expressed as ARE/Nrf2 gene expression fold increase compared to constitutive medium. Values represent means of pentaplicates for all 50/50 combinations and of triplicates for all others. All data were normalized to constitutive medium values; *** *p* < 0.001; ** *p* < 0.01.

The observation of the positive effect of basolateral samples on the ARE/Nrf2 pathway prompted us to evaluate whether combinations could induce some synergistic effects. Among the 27 conditions tested, 7 combinations gave a significant increase in Nrf2/ARE gene expression, ranging between 1.84 (*S. aromaticum*/*V. vinifera*—50/50), 1.70 (*S. aromaticum*/*H. italicum*—50/50), 1.68 (*S. aromaticum*/*H. virginiana—*50/50), 1.58 (*S. aromaticum*/*H. italicum*—35/65), 1.50 (*S. aromaticum*/*H. italicum*—20/80), 1.48 (*S. aromaticum*/*V. vinifera*—35/65) and 1.28 (*H. italicum/V. vinifera*—50/50) (Figure 6).

In order to detect the synergistic effects of these combinations, we compared these measured values with the sum of the increases. The values are presented by each of the extracts tested separately. These results are presented in Table 6. It appears that two combinations, *S. aromaticum/H. italicum* at 35/65 and 20/80 and *S. aromaticum/V. vinifera* (seed) at 50/50 and 35/65, show synergistic effects between 14% and 22.3%.

## 4. Discussion

This study shows that cell-based assays provide a powerful screening system to select natural extracts on the basis of their intracellular antioxidant activity. The Nrf2/ARE assay proved to be sensitive enough to detect antioxidant effects downstream of intestinal transport, opening the way to study the synergistic effects of combinations of metabolites produced in post-intestinal barrier compartments. In a screening campaign carried out in parallel to this study, intestinal epithelial cells have been used in combination with human adipose cells to explore the activities of plant extracts, revealing the relevancy of this method to document the physiological activities of plant extracts [21].

The strong antioxidant effects (µg/mL range) observed in this study are supported by the compounds we could actually detect in the samples used in this study. Some of them are presented in Table 7.

It is remarkable that the synergistic effects could be demonstrated both before and after intestinal transport for three combinations, namely *S. aromaticum/H. italicum*, *S. aromaticum/H. virginiana* and *S. aromaticum/V. vinifera*. The present study provides the first demonstration of such effects. For these four extracts taken individually, the direct and indirect antioxidant effects we observed here are in line with previously published data.

In the case of *S. aromaticum*, for instance, the Nrf2/ARE antioxidant EC_50_ was evaluated at 233 μg/mL in HepG2 cells, the lowest value among the 12 tested extracts (Table 4). This extract also gave EC_50_s in the range of 10 micromolar in the AOP1 free-radical-scavenging assay, both in the Caco-2 and HepG2 models (Table 3). With an increase of 2.78 times the gene expression induced by Nrf2/ARE, the *S. aromaticum* extract also showed the best post-intestinal barrier antioxidant effect of all the tested conditions (Figure 5). It is well-known that clove contains polyphenols, such as hydroxybenzoic acids, hydroxyphenyl propene, hydroxycinnamic acids and eugenol—the major bioactive compound—and gallic acid derivatives, such as hydrolysable tannins [22]. Moreover, clove contains flavonoids (kaempferol, quercetin, etc.) and phenolic acids (salicylic, ferulic, ellagic and caffeic acids, etc.) [23], all known to exert antioxidant effects. The main polyphenols detected in our extract are presented in Table 7. The superiority of flavonoid content and antioxidant activity in crude clove has been observed in comparison with many other crude plants (even *Curcuma longa*) [24]. In accordance with our extract characterization, the clove polyphenols responsible for antioxidant activities have been notably highlighted as quercetin glucoside, isorhamnetin, biflorin and other derivatives [25,26,27]. Specifically, some of these compounds have also demonstrated direct implications and support for the detoxification process, notably through Nrf2 activation [28,29]. In a previous study, an H_2_O_2_ scavenging activity could be detected in cell-free assays of clove bud oil and, to a lesser extent, in clove bud [30]. Another study has demonstrated that water extraction of clove presented a higher yield in extraction of flavonoids and antioxidant activity in vitro, compared to ethanol or ethyl acetate solvent extraction [31]. In streptozotocin-induced diabetic rats, treatment with the bark extract of *S. aromaticum* increased the expression of Nrf2 protein, leading to an upregulation of glyoxalase 1 and a downregulation of the receptor for AGEs [32]. In the present study, the data collected from the HepG2 human liver cells showed high free-radical-quenching and Nrf2 activities. This antioxidant activity of *S. aromaticum* in this cell model is not surprising, as the plant has already proven effective for liver support in other contexts. Clove has been reported for liver protection in traditional uses in India [33]. Many in vivo studies have demonstrated the hepatoprotective effects of clove extracts and crude powder, such as significant toxicity modulation [34], notable antioxidant activities [35] and restoration of normal hepatic safety parameters [36].

Similar to *S. aromaticum*, the *H. italicum* extract gave an Nrf2/ARE EC_50_ of 390.5 μg/mL in Caco-2 cells (Table 4), and an AOP1 EC_50_ of 76.25 μg/mL in the Caco-2 model (Table 3), while showing a significant increase in Nrf2/ARE induction in the post-intestinal barrier compartment. Immortelle is also known to produce antioxidants. Kramberger et al. [37] recently reviewed clinical trials and internal uses for the immortelle aerial part, and they reported that the main active components were phenolic acids, such as caffeic acid and chlorogenic acid, and its derivatives. The same team also reported the presence of flavonoids, namely pinocembrin, quercetin and naringenin [38]. Other identified components were triterpenoids (ursolic acid), acetophenones, phloroglucinols, pyrones and sesquiterpenes, which are all good candidates as antioxidants. The list of main polyphenols we detected in our extract (Table 7) are in line with other data [39]. One of these compounds, leontopodic acid, has demonstrated antioxidant and protective effects against mycotoxin in immunity cells [40]. Interestingly, the analysis of antioxidant capacity by the DPPH radical assay showed that the use of *H. italicum* subspecies (HIT vs. HII) extracted by different procedures led to different results, unrelated to the total phenolic content yield. According to another report, arzanol, a pyrone-phloroglucinol heterodimer, seems to be the most characteristic compound of immortelle that showed a protective effect against lipid oxidation after tert-butyl hydroperoxide (TBH) induction in the plasma membranes of Vero and Caco-2 cells [41]. Scopoletin, another coumarin phenolic compound, was very recently isolated from *H. italicum* and other plants [42]. Interestingly, scopoletin has been shown to exert free-radical-scavenging activity in *Morinda citrifolia* (noni fruit) [43] and activate the Keap1-Nrf2/ARE pathway in SH-SY5Y cells [44].

With an Nrf2/ARE EC_50_ of 634 μg/mL measured in Caco-2 cells (Table 4), and AOP1 EC_50_s of, respectively, 18.58 and 23 μg/mL in the Caco-2 and HepG2 models (Table 3), *H. virginiana* is one of the strongest antioxidant extracts tested in this study. As with the two previously discussed extracts, it also showed a significant Nrf2/ARE induction in the post-intestinal barrier compartment. Witch hazel (*H. virginiana*) has long been used in traditional herbal medicine, and its antioxidant activities have been utilized for decades [45]. The bark polyphenols are mainly hamamelitannin and various proanthocyanidins (PACs). On the other hand, the leaf polyphenols are a mixture of gallotannins and PACs [46]. The main polyphenols specifically detected in our samples are presented in Table 7. The superoxide scavenging properties of hamamelitannin were demonstrated using electron spin resonance (ESR) [47]. Fractions rich in pyrogallol-containing polyphenols, such as proanthocyanidins, gallotannins and gallates, were shown to be active as free-radical scavengers in HAT (hydrogen atom transfer) and SET (single-electron transfer) assays. Additionally, it was found that they are able to protect red blood cells from free radical induced hemolysis [48].

The six different *V. vinifera* extracts used in the present study (three originating from the seed, two from the fruit pomace and one from the leaf) all showed very strong cell antioxidant activities, with AOP1 EC_50_s ranging from 11.62 to 162.2 μg/mL in Caco-2 cells (Figure 2). Notably, the two highest activities, EC_50_s = 11.62 and 20.96 μg/mL, were found in seed extracts after aqueous extraction and purification (Table 1), followed by pomace extracts with EC_50_s = 24.62 and 40.11 μg/mL and the leaf extract with EC_50_ = 76.75 μg/mL. These results are in agreement with several previous in vitro studies that identified seeds as the first reservoir of antioxidant compounds in grape. Grapes are also known to contain large amounts of polyphenols. Flavonoids are mainly located in the skin of the berry, whereas flavan-3-ols (catechins and proanthocyanidins) are present both in the skin and in the seed [49]. For comparison, the antioxidant compounds detected in our sample are presented in Table 7. The total phenolic content and antioxidant capacities (AC) (DPPH, ABTS and FRAP approaches) were determined in a previous study for different parts (pulp, seed and skin) of seven white and fifteen red grape cultivars [50]. Seeds presented the highest ACs, followed by skin and pulp. The same approaches were used in another study to show that the AC was highest in seeds and increased by the altitude [51]. Oligomeric and polymeric procyanidins were also isolated from grape seeds, with the polymeric form presenting the highest ACs, followed by oligomers and monomeric forms of catechins [52]. The trend seems to be different in the context of Nrf2/ARE pathway activation. In this study, we were unable to show any increase in Nrf2/ARE activity in the two *V. vinifera* extracts originating from seeds (Figure 5). However, the leaf extract showed a significant induction of the gene transcription pathway, with an EC_50_ around 661 μg/mL. As a comparison, the work of Esatbeyoglu et al. [53] can be considered here. In this work, the authors have studied the Nrf2 pathway activation of a root extract of *V. vinifera* in the Huh7 human liver hepatoma cell line. They concluded that the extract, at a concentration of 50 μg/mL, significantly induced Nrf2 and its downstream target genes, heme oxygenase-1 (HO-1) and γ-glutamylcysteine synthetase (γ-GCS). Eventually, two studies carried out on rat models showed that the consumption of grapes by hypertensive rats reduced heart failure and increased Nrf2 transcription factor activity [54], while in a model of cardiorenal-injured rats, a combination of silymarin and *V. vinifera* extract synergistically promoted the activation of the Keap1/Nrf2 signaling pathway [55].

This study has brought to light that, among the two main antioxidant approaches we could assay on cell systems, only the ARE/Nrf2 pathway activation is detectable downstream of the intestinal barrier. The Antioxidant Response Element (ARE) is known to regulate a pool of around 600 genes coding for multiple enzymes and regulatory proteins involved in the redox modulation and detoxification processes [56,57]. The ARE/Nrf2 is a regulatory pathway shared by all cells, but its importance has been established in vivo in different pathological contexts [58], such as liver tissues, which are the location of detoxification processes [59,60]; ocular diseases [61], including diseases of the macula, which is widely exposed to UV rays and visible light [62]; erectile dysfunction [63]; neurological disorders [64]; and inflammation [65].

## 5. Conclusions

The selection process we have undertaken in this study allowed us to identify plant extracts with a high value in terms of direct (intracellular free-radical-quenching) or indirect (Nrf2 gene transcription pathway activation) antioxidant effects. We then added an intestinal epithelial barrier step, as this physiological barrier should be considered crucial when studying extracts dedicated to oral use. It was fascinating to discover that only the indirect antioxidant effects could be detected in the basolateral compartments downstream of the barrier, suggesting that antioxidant effects could be transferred from the intestine to the portal vein. Significant differences in indirect antioxidant activities have pointed toward the importance of considering the intestinal passage in the exploration of the potency of oral botanical extracts. Finally, we took advantage of the powerful antioxidant effects of some extracts to test combinations of them, and found the existence of synergistic effects present in both pre and postbarrier compartments. Overall, the combinations using *H. aromaticum*, *V. vinifera*, *H. virginiana* and *H. italicum* should be noticed because of their synergies of action. However, we understand that these effects may well be due to the presence of postbarrier metabolites, which would be interesting to identify to further complete this study.

In a broader perspective, it is important to note that the cell-based technologies used in this study are generic approaches totally open to testing other materials, such as natural extracts, on any available cell models, including stem cells. The only limitation lies in the ability of the active principle to penetrate the cells. Human cell-based approaches are being used more and more as predictive models, providing a bridge between biological activities and human clinical assays without going through the use of expensive, low-throughput, ethically questionable and poor-predictive animal studies [66,67]. With the information obtained by combining cell-based assays and an intestinal absorption system, the present study is, without a doubt, part of this movement.

## Figures and Tables

**Table 1:** List of the 28 natural extracts screened in the study.

Ref	Scientific Name	Common Name	Plant Part	Extraction Process
AV	*Andrographis paniculata*	Green chiretta	aerial part	S/L aqueous extract
AL	*Cinnamomum verum*	Ceylon cinnamon	bark	S/L aqueous extract
BD	*Ficus Carica*	Fig	leaf	S/L aqueous extract
AQ	*Glycyrrhiza glabra*	Licorice	root	S/L aqueous extract
AX	*Hamamelis virginiana*	Hamamelis	aerial part	S/L aqueous extract
AA	*Helichrysum italicum*	Immortelle	aerial part	S/L aqueous extract
AY	*Ilex paraguariensis*	Yerba mate	leaf	S/L aqueous extract
BF	*Lonicera caerulea*	Haskap	fruit	juice extraction
AJ	*Malpighia emarginata*	Acerola	fruit	juice extraction
AZ	*Melissa officinalis*	Lemon balm	leaf	S/L aqueous extract
BE	*Morus nigra*	Black mulberry	leaf	S/L aqueous extract
AU	*Ocimum tenuiflorum*	Holy basil	leaf	S/L aqueous extract
AG	*Olea europaea*	Olive	fruit	juice extraction; absorbent resin purification
AK	*Ribes nigrum*	Blackcurrant	leaf	S/L aqueous extract
AR	*Rosmarinus officinalis*	Rosemary	leaf	S/L aqueous extract
BA	*Salvia officinalis*	Sage	leaf	S/L aqueous extract
AP	*Sambucus nigra*	Elderberry	fruit	juice extraction
AM	*Syzygium aromaticum*	Clove	flower bud	S/L aqueous extract
AS	*Thymus vulgaris*	Thyme	leaf	S/L aqueous extract
AW	*Undaria pinnatifida*	Wakame	algae	S/L aqueous extract
AI	*Vaccinium macrocarpon*	Cranberry	fruit	juice extraction
BB	*Vaccinium myrtillus*	Bilberry	fruit	juice extraction
AN	*Vitis vinifera 1*	Grape	leaf	S/L aqueous extract
AD	*Vitis vinifera 2*	Grape	fruit pomace	S/L aqueous extraction and ethanol purification
AE	*Vitis vinifera 3*	Grape	fruit pomace	S/L aqueous extraction and ethanol purification
AO	*Vitis vinifera 4*	Grape	seed	S/L aqueous extract
AB	*Vitis vinifera 5*	Grape	seed	S/L aqueous extraction; absorbent resin purification
AC	*Vitis vinifera 6*	Grape	seed	S/L aqueous extraction and purification

**Table 2:** Comparisons of AOP1 EC_50_s calculated from dose–response curves obtained with various extract combinations on Caco-2 cells. Theoretical EC_50_s were calculated considering the proportional contribution of each extract and compared with measured EC_50_s. A negative variation indicates a synergistic effect of the tested combination.

Extract Combination	%/%	Theoretical EC_50_ (µg/mL)	Measured EC_50_ (µg/mL)	Var. (%)
*S. aromaticum/H. italicum*	50/50	76.20	39.64	−47.98
	35/65	81.55	104.20	27.78
	20/80	86.90	133.10	53.17
*S. aromaticum/H. virginiana*	50/50	38.47	14.62	−61.99
	35/65	32.49	31.60	−2.75
	20/80	26.52	29.30	10.47
*S. aromaticum/V. vinifera 5*	50/50	40.76	12.74	−68.74
	35/65	35.48	31.30	−11.77
	20/80	30.19	31.70	4.99
*H. virginiana/V. vinifera 5*	50/50	20.86	20.07	−3.76
	40/60	21.31	3.30	−84.52

**Table 3:** Comparison of AOP1 EC_50_s evaluated in Caco-2 and HepG2 cells for the 12 natural extracts selected for the intestinal barrier transport study. Extracts were selected according to the best EC_50_s obtained with AOP1 assay. In order to increase the diversity of tested extracts, some *Vitis vinifera* extracts were removed from the list because of redundancy with each other. ND = not determined (no full effect observed).

Sample	EC_50_ (µg/mL) Caco-2	EC50 (µg/mL) HepG2
*V. vinifera 6*	11.62	15.74
*H. virginiana*	18.58	23.00
*V. vinifera 5*	20.96	16.76
*S. aromaticum*	57.78	46.73
*H. italicum*	76.25	ND
*V. vinifera 1*	76.75	59.78
*V. myrtillus*	80.65	308.20
*I. paraguariensis*	95.43	131.40
*R. nigrum*	97.77	143.30
*C. verum*	102.10	52.09
*R. officinalis*	169.90	174.70
*M. officinalis*	200.50	248.20

**Table 4:** ARE/Nrf2 EC_50_s established in HepG2 cells for the 12 natural extracts selected for the Caco-2 intestinal barrier transport study. ND = not determined; R^2^ = determination coefficient.

Sample	EC_50_ (µg/mL) HepG2	R^2^
*V. vinifera 6*	ND	ND
*H. virginiana*	≈634.0	0.8577
*V. vinifera 5*	ND	ND
*S. aromaticum*	233.4	0.9723
*H. italicum*	390.5	0.9387
*V. vinifera 1*	≈661.1	0.9949
*V. myrtillus*	2636.0	0.9403
*I. paraguariensis*	587.6	0.7842
*R. nigrum*	2878.0	0.9561
*C. verum*	444.6	0.6420
*R. officinalis*	≈630.8	0.9061
*M. officinalis*	2873.0	0.9947

**Table 5:** TEER evaluation. Transepithelial Electrical Resistances (TEER cell monolayer—TEER supporting filter without cells), expressed in Ω × cm^2^, measured on Transwells, before (t = 0) and after (t = 4 h) sample exposure to epithelial monolayer cell model.

Sample	TEER (Ω, t = 0)	TEER (Ω, t = 4 h)	Variation (%)
*V. vinifera 6*	679	457	32.76
*H. virginiana*	798	765	4.08
*V. vinifera 5*	756	505	33.19
*S. aromaticum*	765	475	37.91
*H. italicum*	538	415	22.79
*V. vinifera 1*	847	753	11.12
*V. myrtillus*	761	632	16.98
*I. paraguariensis*	756	686	9.26
*R. nigrum*	580	398	31.32
*C. verum*	831	707	14.94
*R. officinalis*	748	718	3.90
*M. officinalis*	841	648	22.89

**Table 6:** Synergistic effects on ARE/Nrf2 gene expression of post-intestinal barrier compartment combinations. Combin. FI = gene expression fold increase (FI) measure for the combination; S indep. FI = sum of the increase in gene expression fold increase (FI) measures of each of the two extracts taken independently as depicted in Figure 6; Var. (%) = variation in percentage of the two values. A positive percentage value indicates a synergistic effect.

Extract Combination	%/%	Σ Indep. FI	Combin. FI	Var. (%)
*S. aromaticum/H. italicum*	50/50	1.82	1.70	−7.06
	35/65	1.34	1.58	15.19
	20/80	1.29	1.50	14.00
*S. aromaticum/H. virginiana*	50/50	1.65	1.68	1.79
*S. aromaticum/V. vinifera 5*	50/50	1.52	1.84	17.39
	35/65	1.15	1.48	22.30
*H. italicum/V. vinifera 5*	50/50	1.22	1.28	4.69

**Table 7:** Main polyphenols detected in some of the extracts used in this study.

Extract	Compounds
*S. aromaticum*	gallic acid (and gallotannins), quercetin glucoside, isorhamnetin, eugenol, biflorin, and derivatives
*H. virginiana*	gallic acid and gallotannins, catechin, proanthocyanidin B dimer and chlorogenic acid
*V. Vinifera 5*	catechin, epicatechin, epicatechin gallate, gallic acid and proanthocyanidin B dimer, trimer, tetramer
*H. italicum*	dicaffeoylquinic acid, leontopodic acid, and myricetin and quercetin derivatives

## Data Availability

Data is contained within the article and supplementary material.

## Supplementary Materials

The following supporting information can be downloaded at: https://www.mdpi.com/article/10.3390/antiox11030565/s1, Figure S1: AOP1 data on Caco-2 cells; Figure S2: AOP1 on HepG2 cells (pre-apical and basolateral compartments).
