# Supplementary material for: Cell-Based Antioxidant Properties and Synergistic Effects of Natural Plant and Algal Extracts Pre and Post Intestinal Barrier Transport
Source: Antioxidants (Basel). 2022 Mar 16;11(3):565. doi: 10.3390/antiox11030565 (PMC8944961; doi:10.3390/antiox11030565)
Supplement: Supplementary file 1 [file antioxidants-11-00565-s001.zip › antioxidants-1602102-supplementary.pdf]

Supplementary data - Figure S1 – AOP1 data on CaCo2 cells

Sample AA

Assay : AOP 1
Cellular model : CaCo2

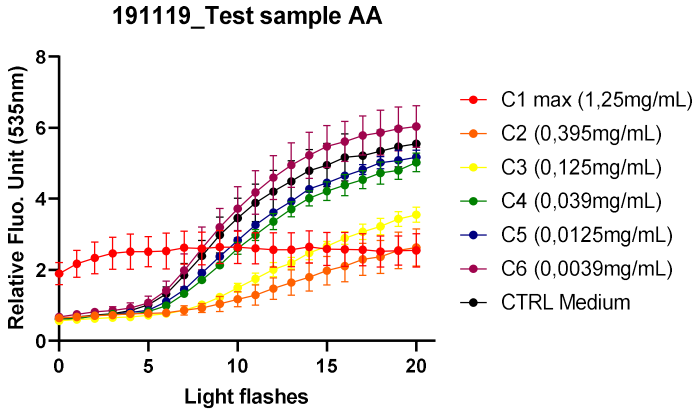

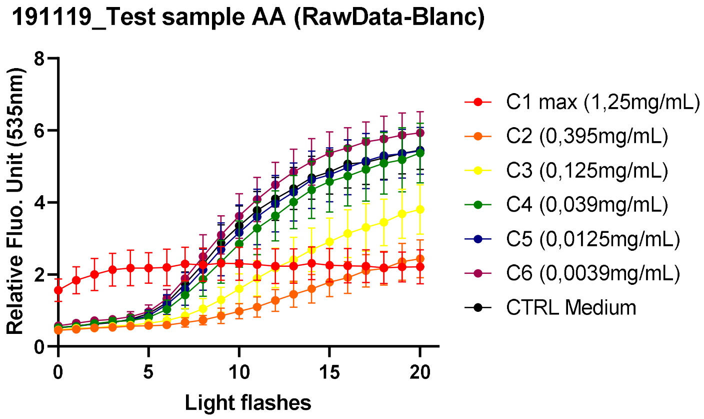

EC_{90} = 309.4 µg/ml
EC_{50} = 76.25 µg/ml
EC_{10} = 18.79 µg/ml
R^2 = 0.9998

Antioxidant index (normalized, max = 1000):
C2 : 748
C3 : 546
C4 : 210
C5 : 30
C6 : -4

Antioxidant index :

$_0\int^{20} RFU_n$ control - $_0\int^{20} RFU_n$ sample

Normalized antioxidant index :

1000 – 1000 ($_0\int^{20} RFU_n$ sample /$_0\int^{20} RFU_n$ control)

Graph legends :

R^2 = Determination coefficient
EC_{50} = 50% efficacy concentration
Error bars correspond to +/- SD value of triplicates (3 wells)

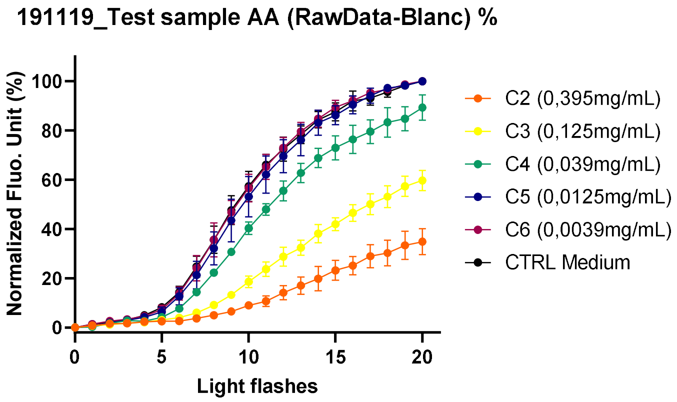

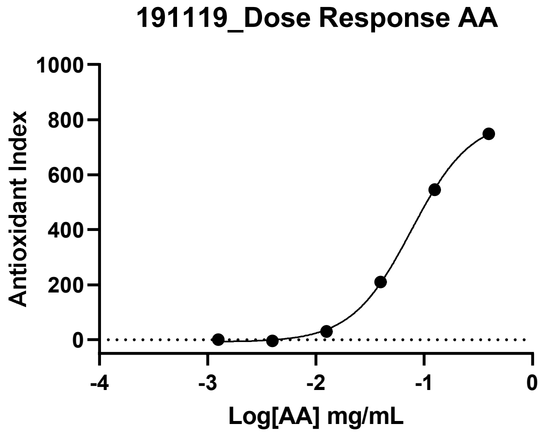

Sample AB

Assay : AOP 1
Cellular model : CaCo2

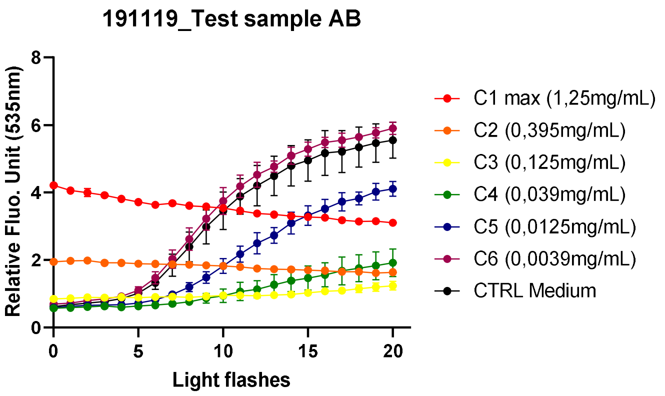

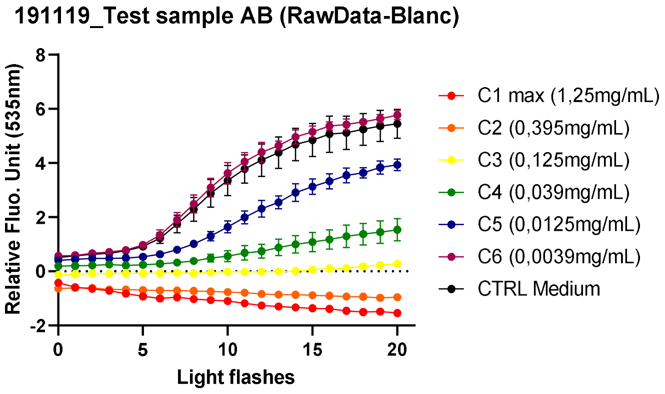

EC_{90} = 48.52 µg/ml
EC_{50} = 20.96 µg/ml
EC_{10} = 9.05 µg/ml
R^2 = 0.9998

Antioxidant index (normalized, max = 1000):
C3 : 932
C4 : 788
C5 : 189
C6 : -5

Antioxidant index :

$\int_0^{20} RFU_n$ control - $\int_0^{20} RFU_n$ sample

Normalized antioxidant index :

1000 – 1000 ($\int_0^{20} RFU_n$ sample / $\int_0^{20} RFU_n$ control)

Graph legends :

R^2 = Determination coefficient
EC_{50} = 50% efficacy concentration
Error bars correspond to +/- SD value of triplicates (3 wells)

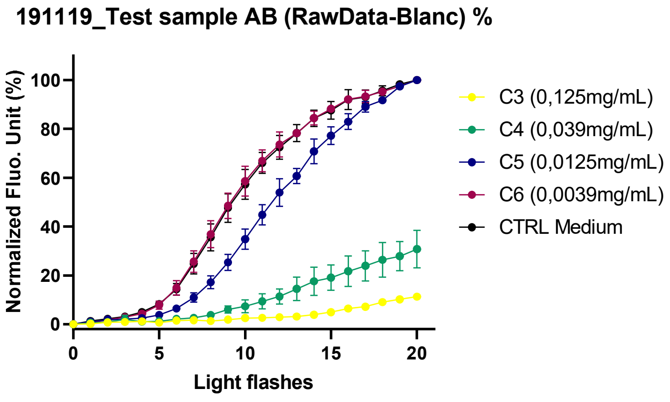

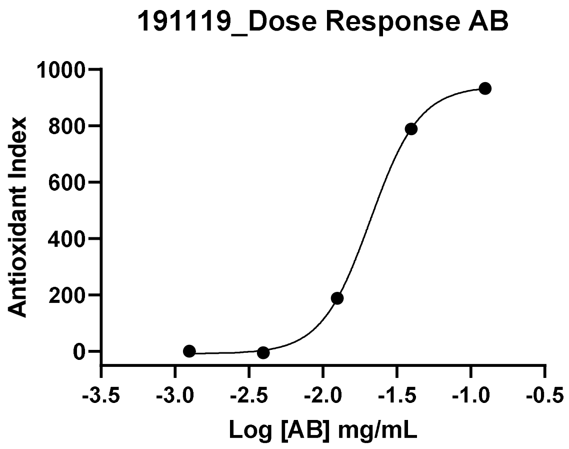

Sample AC

Assay : AOP 1
Cellular model : CaCo2

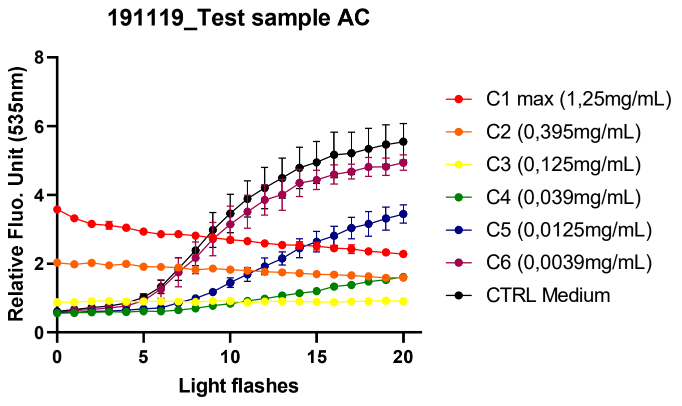

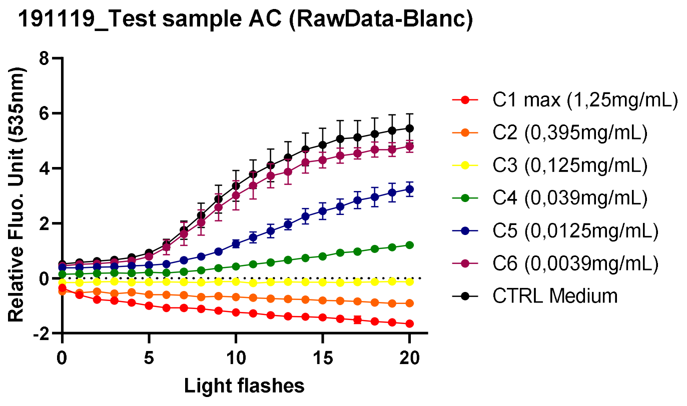

EC_{90} = 29.49 µg/ml
EC_{50} = 11.62 µg/ml
EC_{10} = 4.58 µg/ml
R^2 = 0.9900

Antioxidant index (normalized, max = 1000):
C3 : 992
C4 : 841
C5 : 513
C6 : -13

Antioxidant index :

$_0\int^{20} RFU_n$ control - $_0\int^{20} RFU_n$ sample

Normalized antioxidant index :

1000 – 1000 ($_0\int^{20} RFU_n$ sample /$_0\int^{20} RFU_n$ control)

Graph legends :

R^2 = Determination coefficient
EC_{50} = 50% efficacy concentration
Error bars correspond to +/- SD value of triplicates (3 wells)

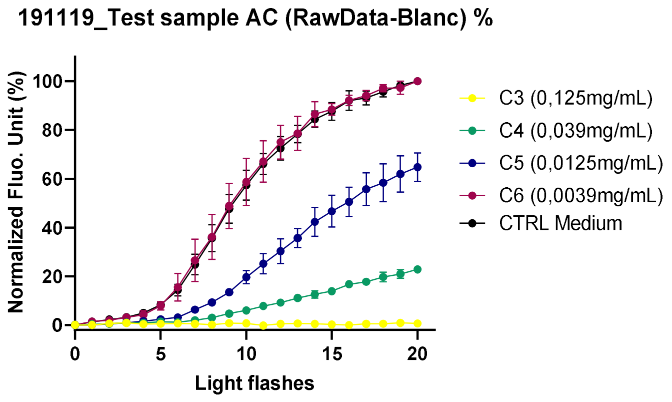

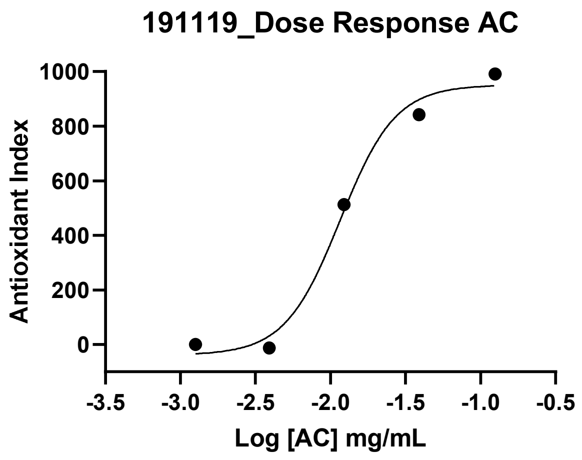

Sample AD

Assay : AOP 1
Cellular model : CaCo2

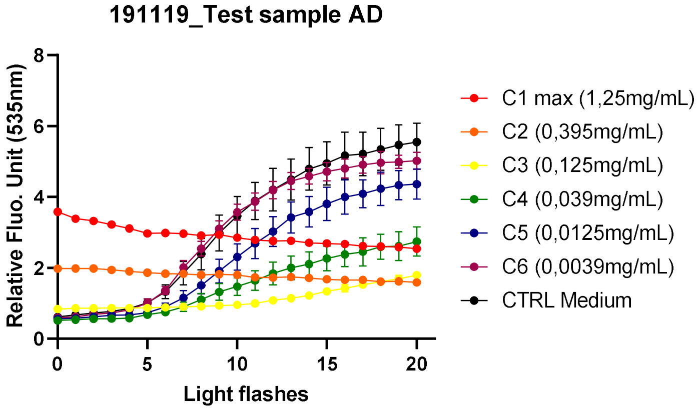

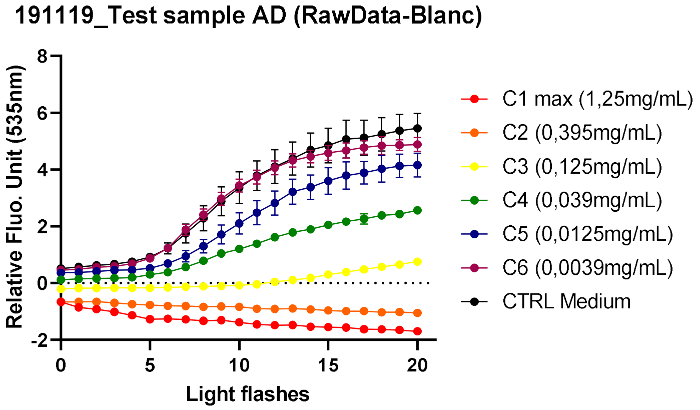

EC_{90} = 137.1 µg/ml
EC_{50} = 40.11 µg/ml
EC_{10} = 11.74 µg/ml
R^2 = 0.9915

Antioxidant index (normalized, max = 1000):
C3 : 874
C4 : 464
C5 : 86
C6 : -87

Antioxidant index :

$_0\int^{20}$ RFU_n control - $_0\int^{20}$ RFU_n sample

Normalized antioxidant index :

1000 – 1000 ($_0\int^{20}$ RFU_n sample /$_0\int^{20}$ RFU_n control)

Graph legends :

R^2 = Determination coefficient
EC_{50} = 50% efficacy concentration
Error bars correspond to +/- SD value of triplicates (3 wells)

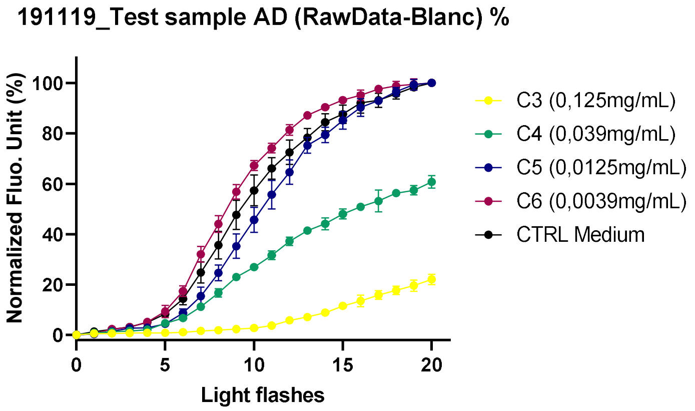

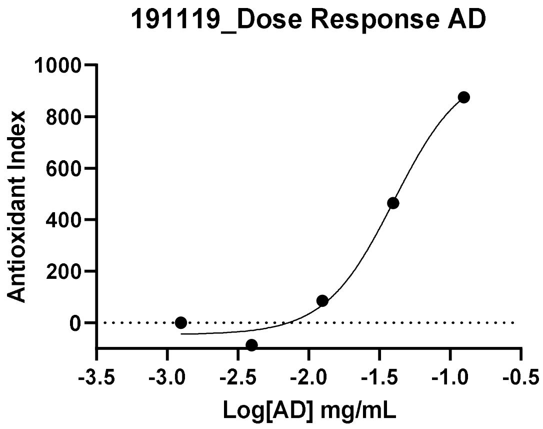

Sample AE	<u>Assay</u> : AOP 1<br><u>Cellular model</u> : CaCo2

191119_Test sample AE

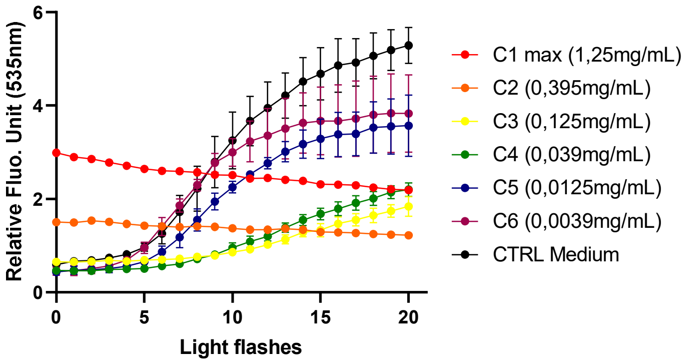

191119_Test sample AE (RawData-Blanc)

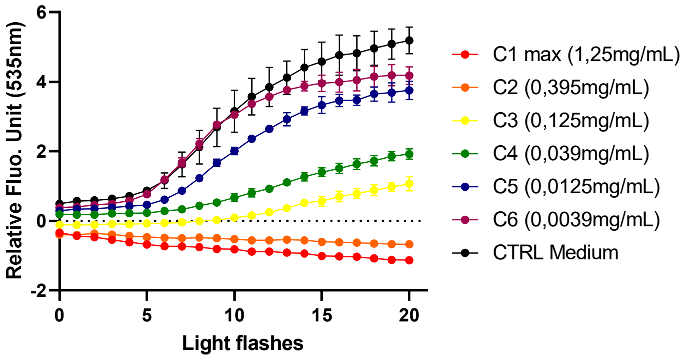

EC_{90} = 56.15 µg/ml
EC_{50} = 24.62 µg/ml
EC_{10} = 10.79 µg/ml
R^2 = 0.9935

Antioxidant index (normalized, max = 1000):
C3 : 797
C4 : 618
C5 : 83
C6 : -84

<u>**Antioxidant index**</u> **:**

$_0\int^{20}$ RFU_n control - $_0\int^{20}$ RFU_n sample

<u>**Normalized antioxidant index**</u> **:**

1000 – 1000 ($_0\int^{20}$ RFU_n sample /$_0\int^{20}$ RFU_n control)

<u>**Graph legends**</u> **:**

R^2 = Determination coefficient
EC_{50} = 50% efficacy concentration
Error bars correspond to +/- SD value of triplicates (3 wells)

191119_Test sample AE (RawData-Blanc) %

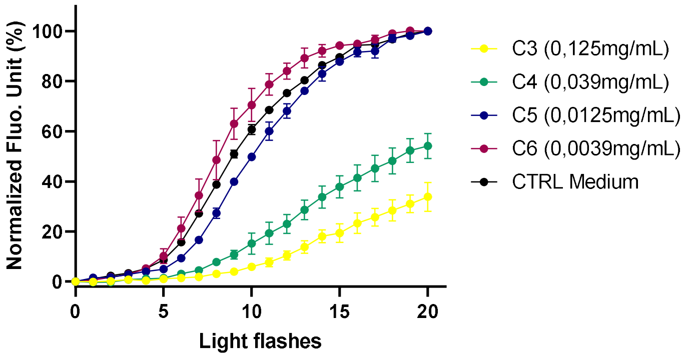

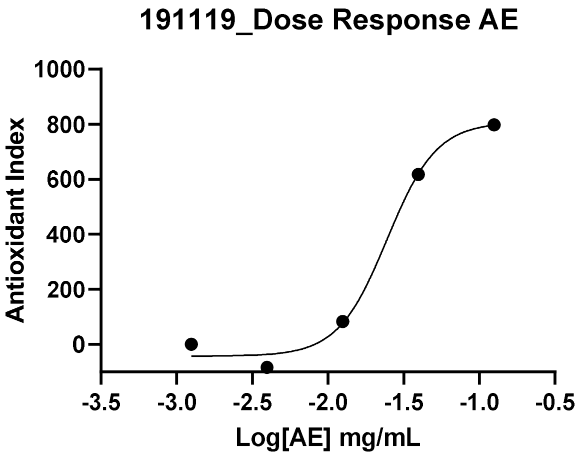

Sample AF

Assay : AOP 1
Cellular model : CaCo2

191119_Test sample AF

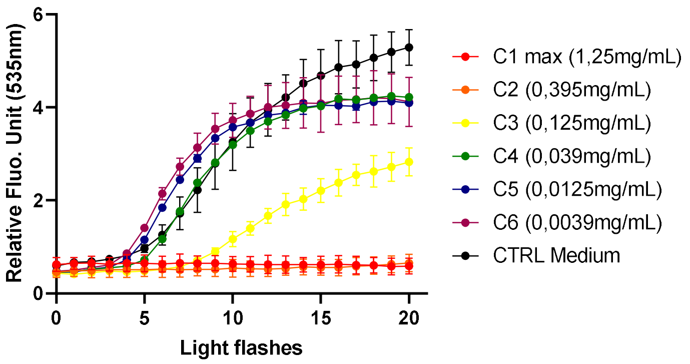

191119_Test sample AF (RawData-Blanc)

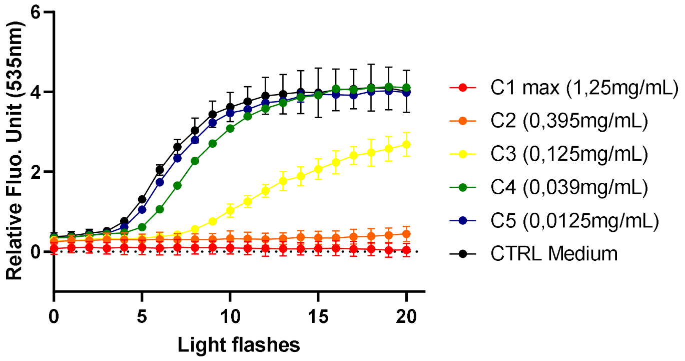

EC_{90} = 227.4 µg/ml
EC_{50} = 89.26 µg/ml
EC_{10} = 35.04 µg/ml
R^2 = 0.9991

Antioxidant index (normalized, max = 1000):
C1 : 995
C2 : 983
C3 : 696
C4 : 144
C5 : 50

Antioxidant index :

$_0\int^{20} RFU_n$ control - $_0\int^{20} RFU_n$ sample

Normalized antioxidant index :

1000 – 1000 ($_0\int^{20} RFU_n$ sample /$_0\int^{20} RFU_n$ control)

Graph legends :

R^2 = Determination coefficient
EC_{50} = 50% efficacy concentration
Error bars correspond to +/- SD value of triplicates (3 wells)

191119_Test sample AF (RawData-Blanc) %

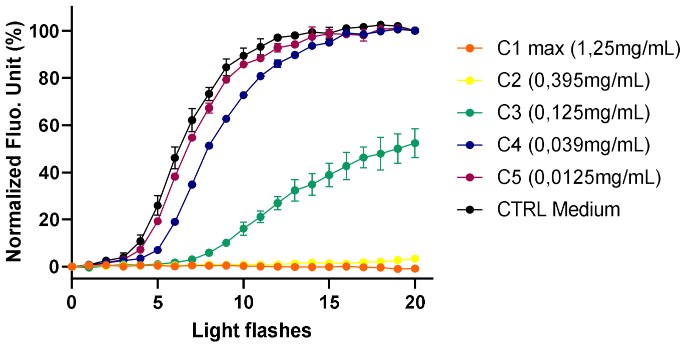

191119_Dose Response AF

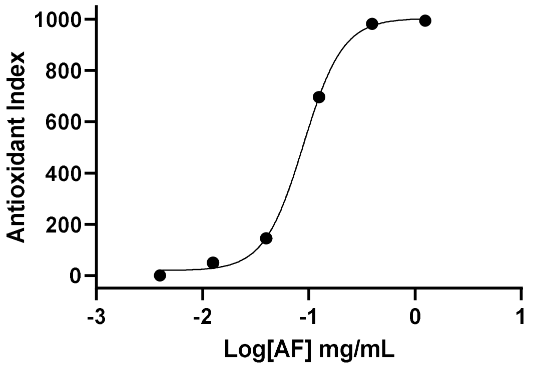

Sample AG

Assay : AOP 1
Cellular model : CaCo2

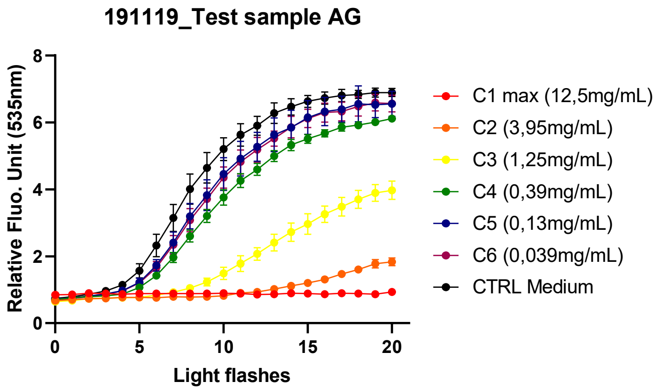

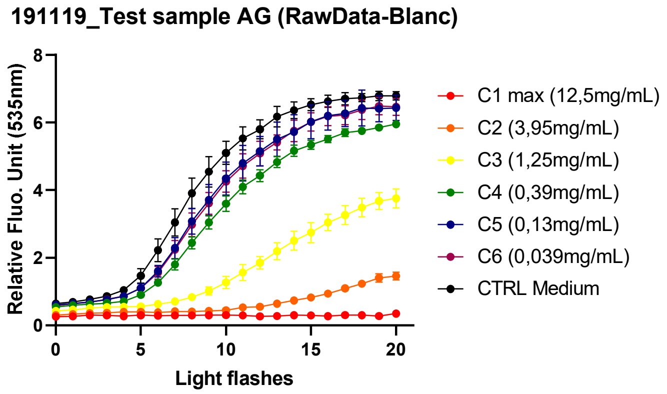

EC_{90} = 2877 µg/ml
EC_{50} = 1037 µg/ml
EC_{10} = 374 µg/ml
R^2 = 0.9949

Antioxidant index (normalized, max = 1000):
C1 : 990
C2 : 898
C3 : 614
C4 : 133
C5 : 76
C6 : 85

Antioxidant index :

$_0\int^{20}$ RFU_n control - $_0\int^{20}$ RFU_n sample

Normalized antioxidant index :

1000 – 1000 ($_0\int^{20}$ RFU_n sample /$_0\int^{20}$ RFU_n control)

Graph legends :

R^2 = Determination coefficient
EC_{50} = 50% efficacy concentration
Error bars correspond to +/- SD value of triplicates (3 wells)

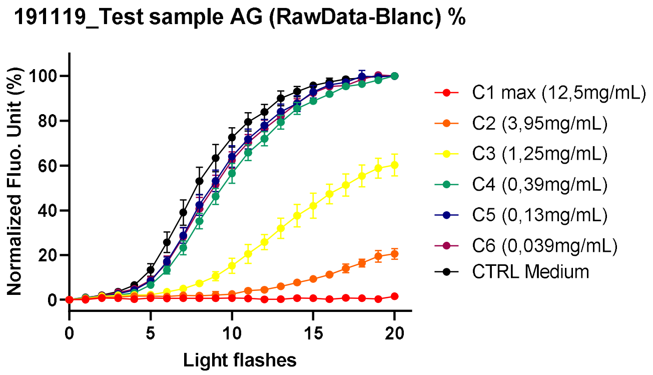

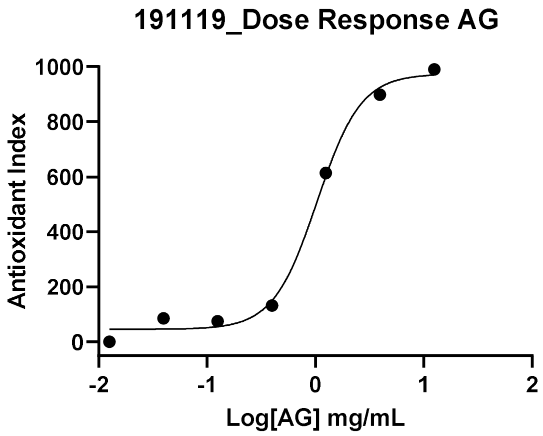

Sample AI

<u>Assay</u> : AOP 1
<u>Cellular model</u> : CaCo2

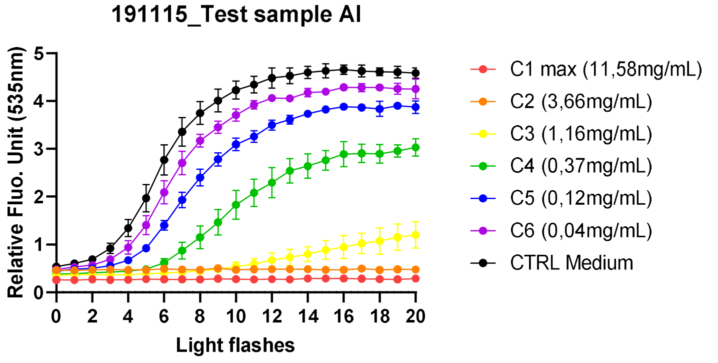

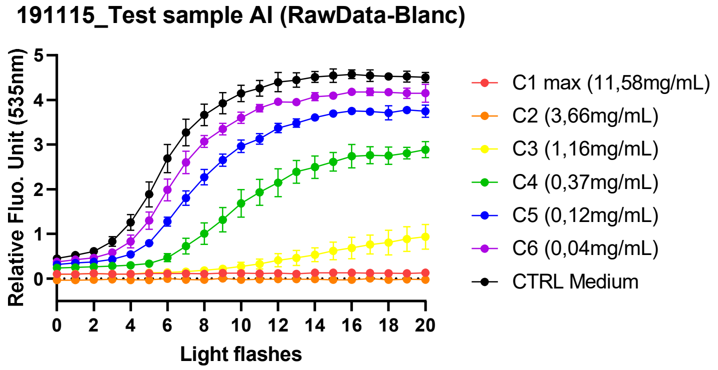

EC_{90} = 1516 µg/ml
EC_{50} = 566.5 µg/ml
EC_{10} = 211.7 µg/ml
R^2 = 0.9949

Antioxidant index (normalized, max = 1000):
C1 : 992
C2 : 992
C3 : 854
C4 : 297
C5 : 134
C6 : 57

<u>Antioxidant index</u> :

$_0\int^{20}$ RFU_n control - $_0\int^{20}$ RFU_n sample

<u>Normalized antioxidant index</u> :

1000 – 1000 ($_0\int^{20}$ RFU_n sample /$_0\int^{20}$ RFU_n control)

<u>Graph legends</u> :

R^2 = Determination coefficient
EC_{50} = 50% efficacy concentration
Error bars correspond to +/- SD value of triplicates (3 wells)

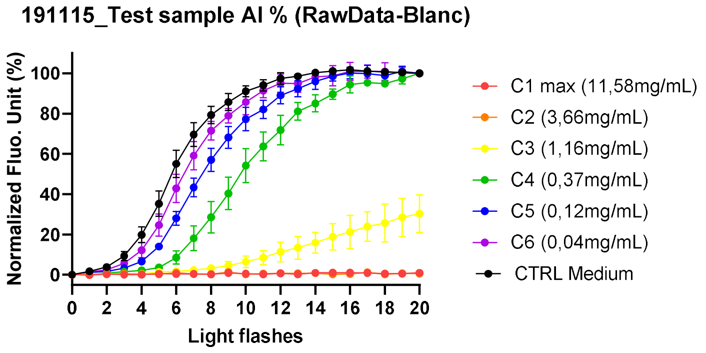

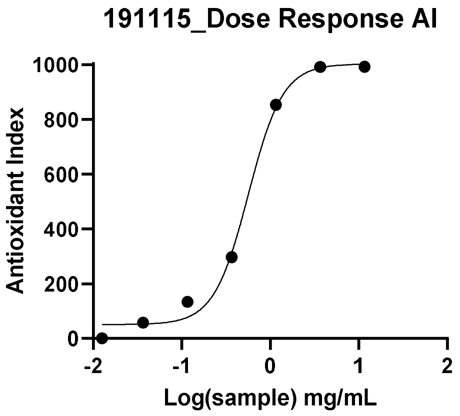

Sample AJ

Assay : **AOP 1**
Cellular model : **CaCo2**

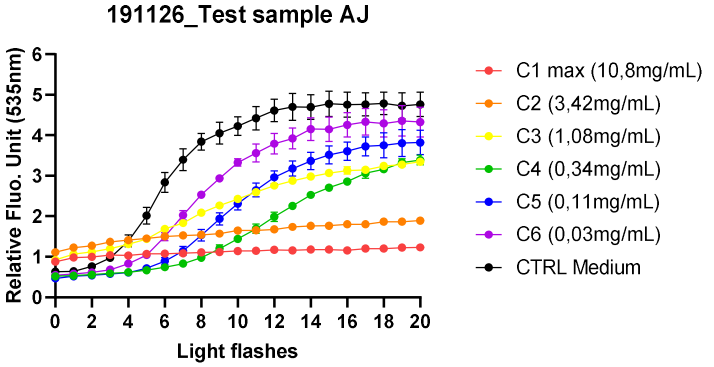

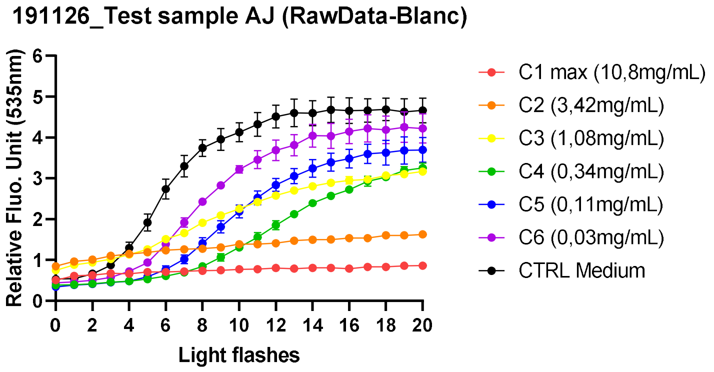

EC_{90} = ND
EC_{50} = ND
EC_{10} = ND
R^2 = 0.8405

Antioxidant index (normalized, max = 1000):
C4 : 510
C5 : 254
C6 : 138

Antioxidant index :

$_0\int^{20}$ RFU_n control - $_0\int^{20}$ RFU_n sample

Normalized antioxidant index :

1000 – 1000 ($_0\int^{20}$ RFU_n sample /$_0\int^{20}$ RFU_n control)

Graph legends :

R^2 = Determination coefficient
EC_{50} = 50% efficacy concentration
Error bars correspond to +/- SD value of triplicates (3 wells)

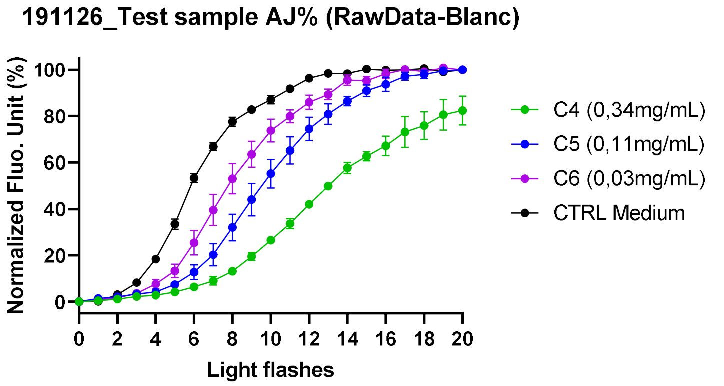

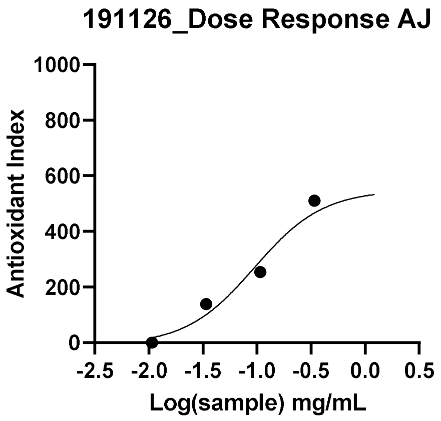

Sample AK

Assay : AOP 1
Cellular model : CaCo2

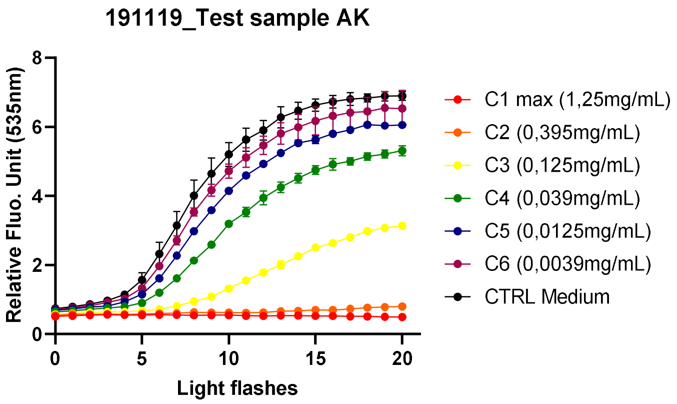

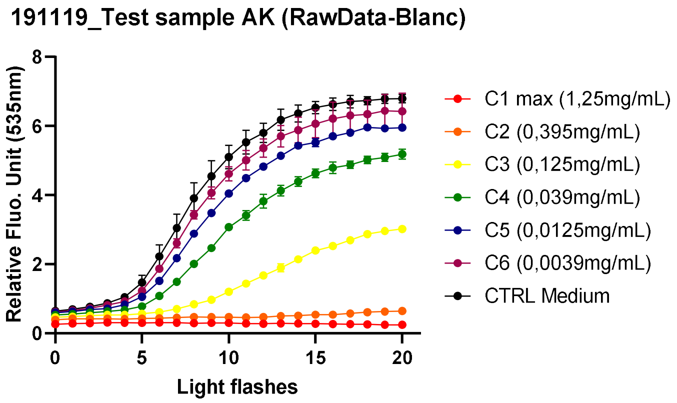

EC_{90} = 284.4 µg/ml
EC_{50} = 97.77 µg/ml
EC_{10} = 33.61 µg/ml
R^2 = 0.9985

Antioxidant index (normalized, max = 1000):
C1 : 991
C2 : 963
C3 : 636
C4 : 156
C5 : 69
C6 : 33

Antioxidant index :

$_0\int^{20}$ RFU_n control - $_0\int^{20}$ RFU_n sample

Normalized antioxidant index :

1000 – 1000 ($_0\int^{20}$ RFU_n sample /$_0\int^{20}$ RFU_n control)

Graph legends :

R^2 = Determination coefficient
EC_{50} = 50% efficacy concentration
Error bars correspond to +/- SD value of triplicates (3 wells)

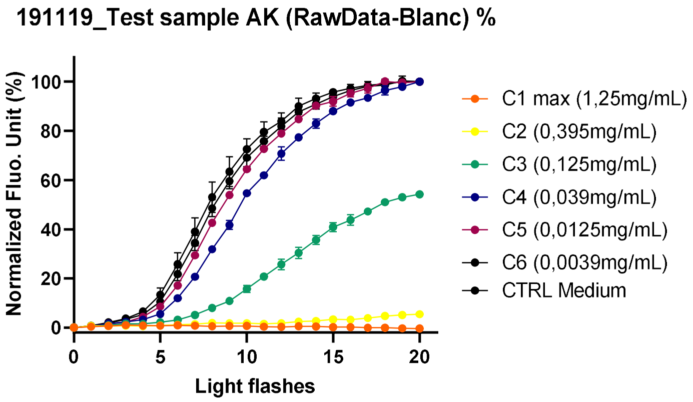

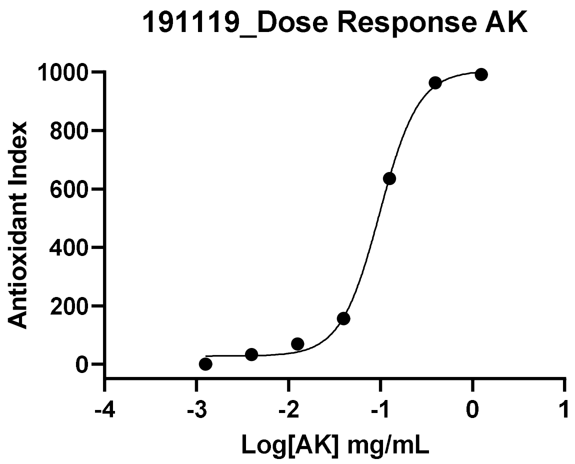

Sample AL

Assay : AOP 1
Cellular model : CaCo2

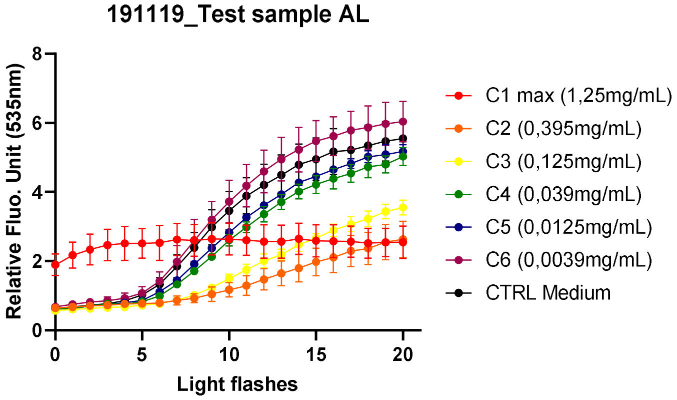

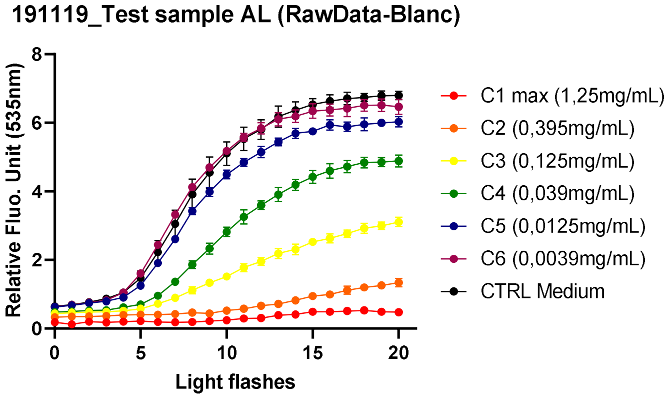

EC_{90} = 390.3 µg/ml
EC_{50} = 102.1 µg/ml
EC_{10} = 26.68 µg/ml
R^2 = 0.9986

Antioxidant index (normalized, max = 1000):
C1 : 952
C2 : 875
C3 : 553
C4 : 150
C5 : 13
C6 : -51

Antioxidant index :

$_0\int^{20}$ RFU_n control - $_0\int^{20}$ RFU_n sample

Normalized antioxidant index :

1000 – 1000 ($_0\int^{20}$ RFU_n sample /$_0\int^{20}$ RFU_n control)

Graph legends :

R^2 = Determination coefficient
EC_{50} = 50% efficacy concentration
Error bars correspond to +/- SD value of triplicates (3 wells)

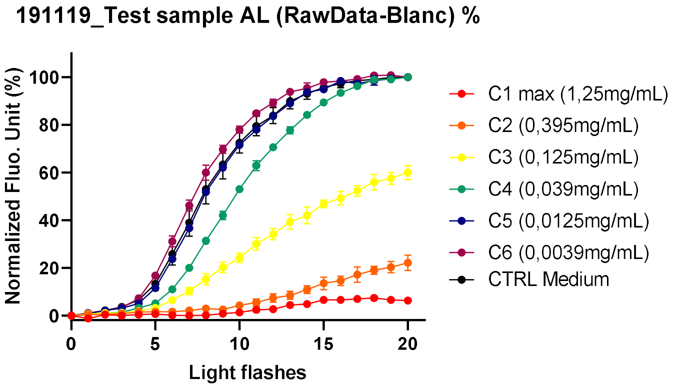

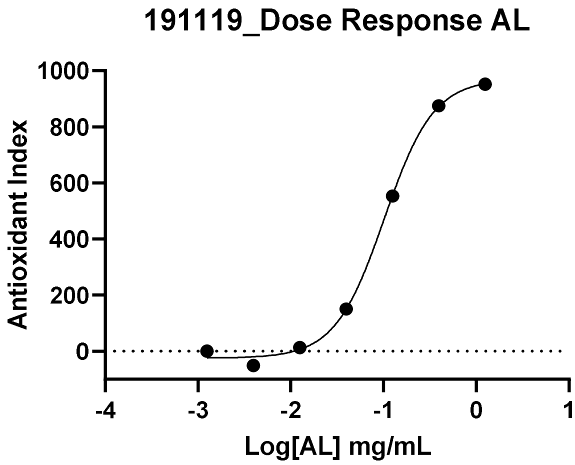

Sample AM

Assay : AOP 1
Cellular model : CaCo2

191115_Test sample AM

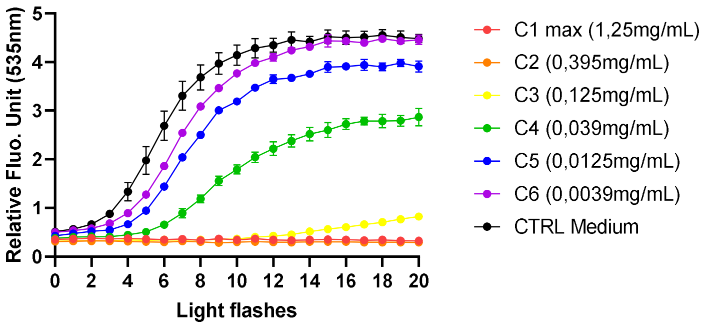

191115_Test sample AM (RawData-Blanc)

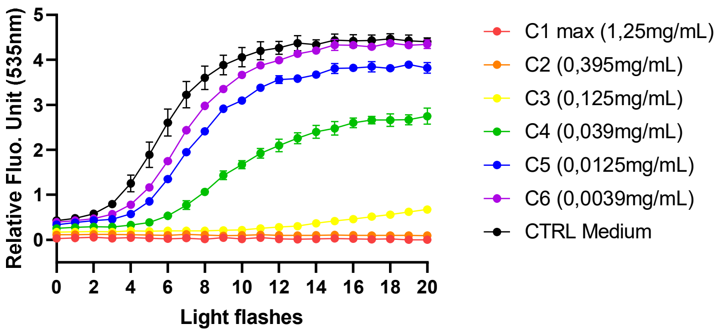

EC_{90} = 118 µg/ml
EC_{50} = 57.78 µg/ml
EC_{10} = 28.29 µg/ml
R^2 = 0.9947

Antioxidant index (normalized, max = 1000):
C1 : 994
C2 : 995
C3 : 923
C4 : 276
C5 : 114
C6 : 96

Antioxidant index :

$_0\int^{20}$ RFU_n control - $_0\int^{20}$ RFU_n sample

Normalized antioxidant index :

1000 – 1000 ($_0\int^{20}$ RFU_n sample /$_0\int^{20}$ RFU_n control)

Graph legends :

R^2 = Determination coefficient
EC_{50} = 50% efficacy concentration
Error bars correspond to +/- SD value of triplicates (3 wells)

191115_Test sample AM % (RawData-Blanc)

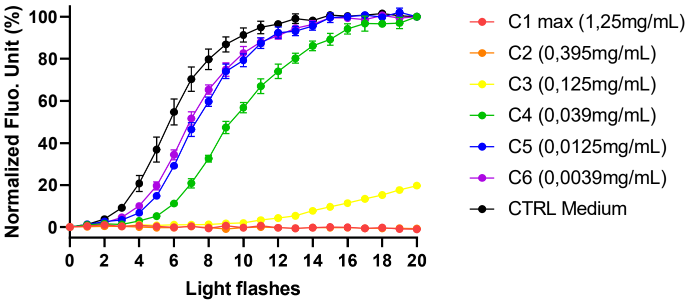

191115_Dose Response AM

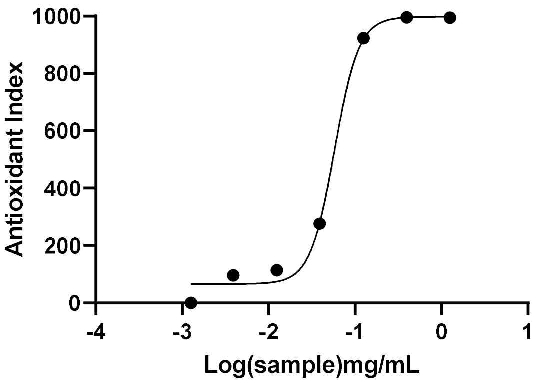

Sample AN

Assay : AOP 1
Cellular model : CaCo2

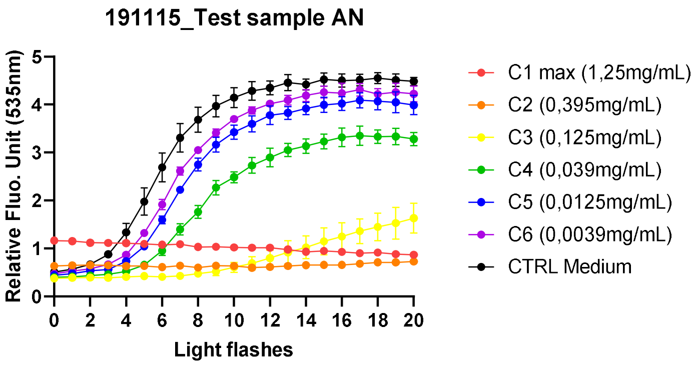

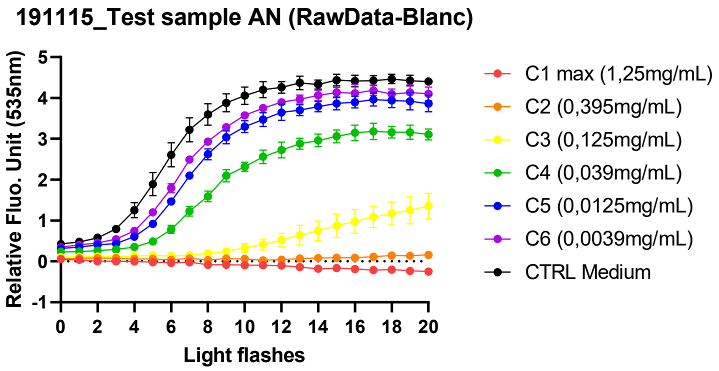

EC_{90} = 167.5 µg/ml
EC_{50} = 76.75 µg/ml
EC_{10} = 35.16 µg/ml
R^2 = 0.9959

Antioxidant index (normalized, max = 1000):
C2 : 989
C3 : 808
C4 : 167
C5 : 88
C6 : 65

Antioxidant index :

$_0\int^{20}$ RFU_n control - $_0\int^{20}$ RFU_n sample

Normalized antioxidant index :

1000 – 1000 ($_0\int^{20}$ RFU_n sample /$_0\int^{20}$ RFU_n control)

Graph legends :

R^2 = Determination coefficient
EC_{50} = 50% efficacy concentration
Error bars correspond to +/- SD value of triplicates (3 wells)

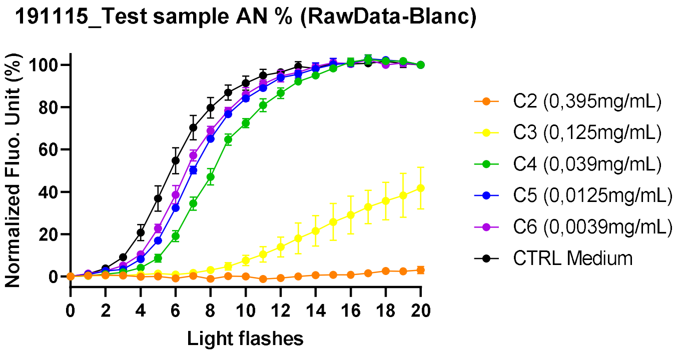

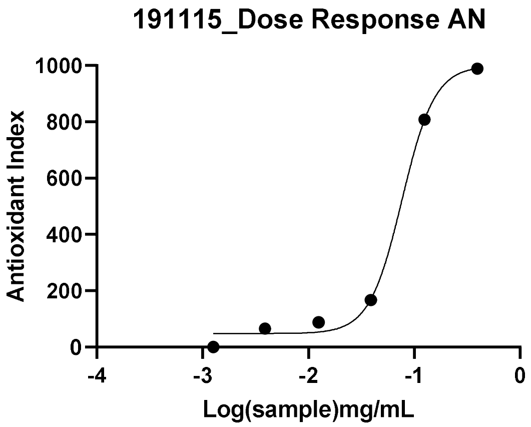

Sample AO	Assay : AOP 1<br>Cellular model : CaCo2

191115_Test sample AO

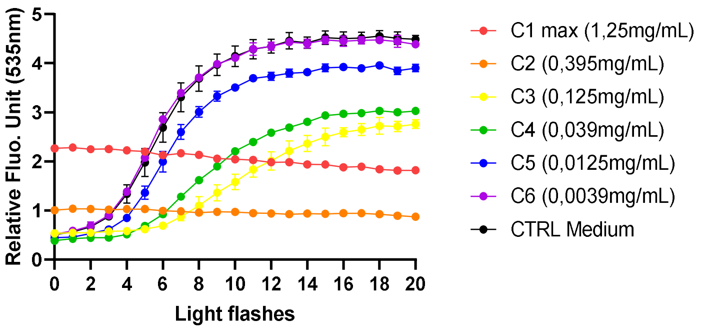

191115_Test sample AO (RawData-Blanc)

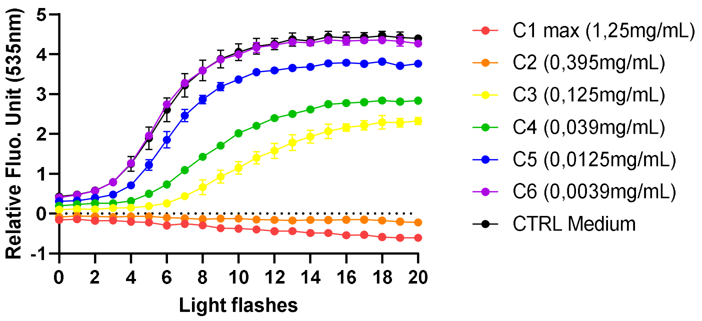

EC_{90} = 328.7 µg/ml
EC_{50} = 162.2 µg/ml
EC_{10} = 80.08 µg/ml
R^2 = 0.9764

Antioxidant index (normalized, max = 1000):
C2 : 971
C3 : 325
C4 : 192
C5 : 46
C6 : -27

Antioxidant index :

$_0\int^{20}$ RFU$_n$ control - $_0\int^{20}$ RFU$_n$ sample

Normalized antioxidant index :

1000 – 1000 ($_0\int^{20}$ RFU$_n$ sample /$_0\int^{20}$ RFU$_n$ control)

Graph legends :

R^2 = Determination coefficient
EC_{50} = 50% efficacy concentration
Error bars correspond to +/- SD value of triplicates (3 wells)

191115_Test sample AO % (RawData-Blanc)

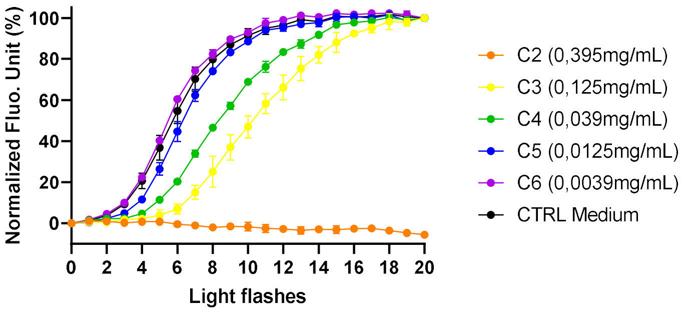

191115_Dose Response AO

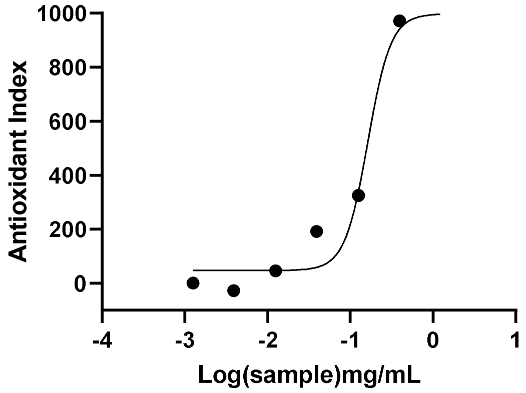

Sample AP

Assay : AOP 1
Cellular model : CaCo2

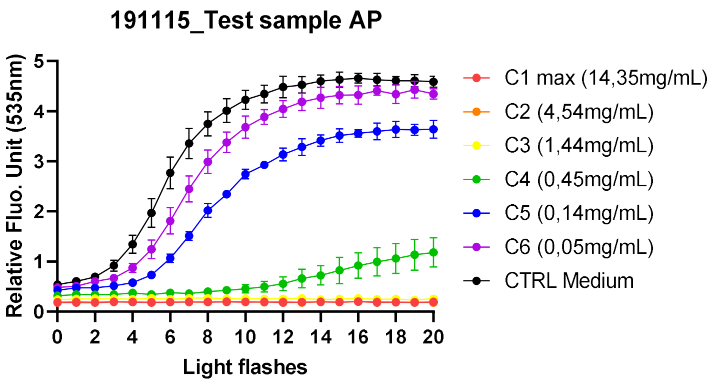

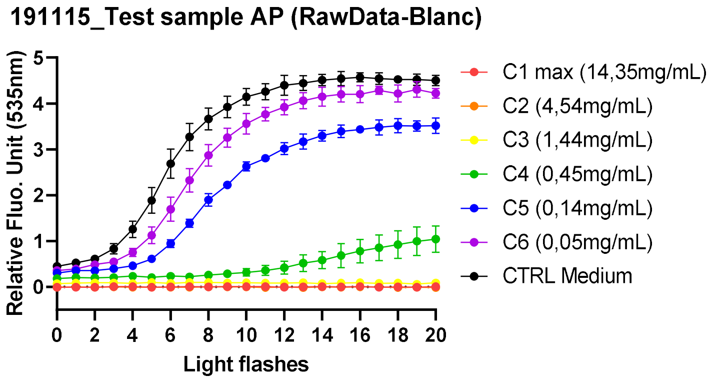

EC_{90} = 516.4 µg/ml
EC_{50} = 240.1 µg/ml
EC_{10} = 111.7 µg/ml
R^2 = 0.9948

Antioxidant index (normalized, max = 1000):
C1 : 996
C2 : 998
C3 : 992
C4 : 872
C5 : 225
C6 : 122

Antioxidant index :

$_0\int^{20}$ RFU_n control - $_0\int^{20}$ RFU_n sample

Normalized antioxidant index :

1000 – 1000 ($_0\int^{20}$ RFU_n sample /$_0\int^{20}$ RFU_n control)

Graph legends :

R^2 = Determination coefficient
EC_{50} = 50% efficacy concentration
Error bars correspond to +/- SD value of triplicates (3 wells)

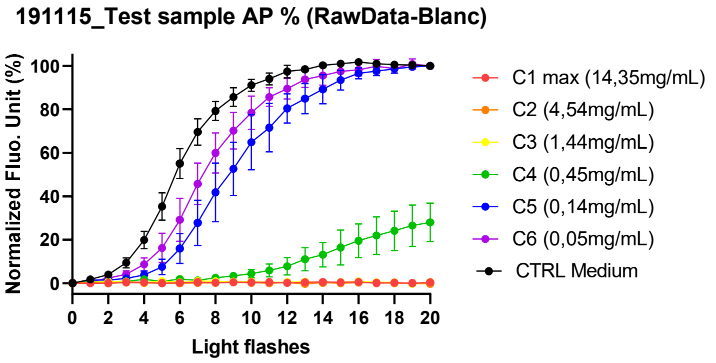

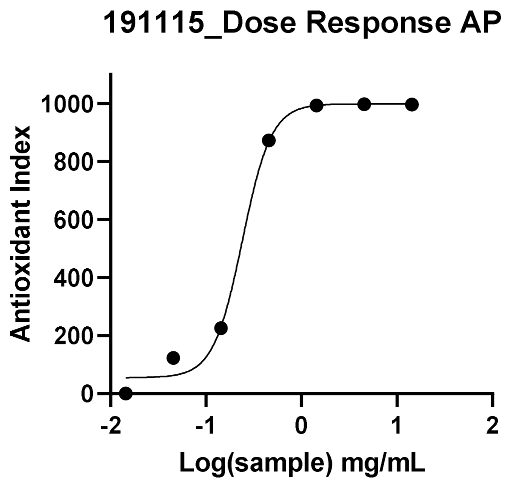

Sample AQ

Assay : AOP 1
Cellular model : CaCo2

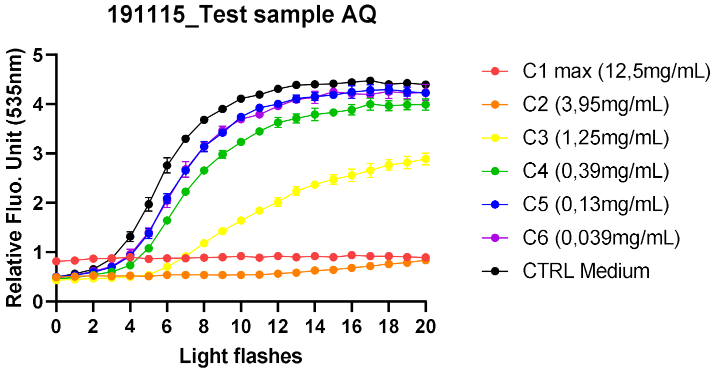

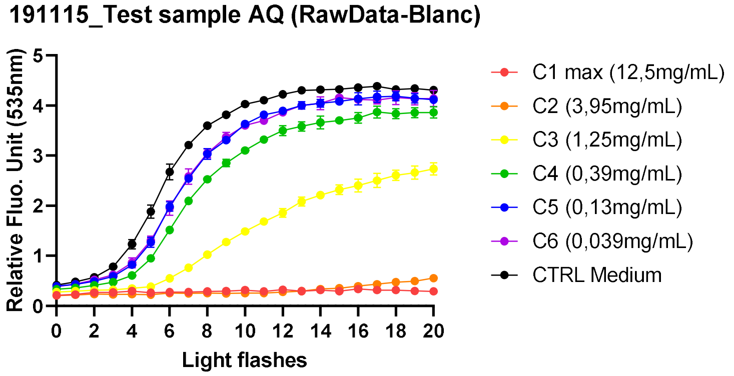

EC_{90} = 3019 µg/ml
EC_{50} = 1176 µg/ml
EC_{10} = 457.8 µg/ml
R^2 = 0.9961

Antioxidant index (normalized, max = 1000):
C1 : 969
C2 : 962
C3 : 545
C4 : 129
C5 : 69
C6 : 73

Antioxidant index :

$_0\int^{20} RFU_n$ control - $_0\int^{20} RFU_n$ sample

Normalized antioxidant index :

1000 – 1000 ($_0\int^{20} RFU_n$ sample /$_0\int^{20} RFU_n$ control)

Graph legends :

R^2 = Determination coefficient
EC_{50} = 50% efficacy concentration
Error bars correspond to +/- SD value of triplicates (3 wells)

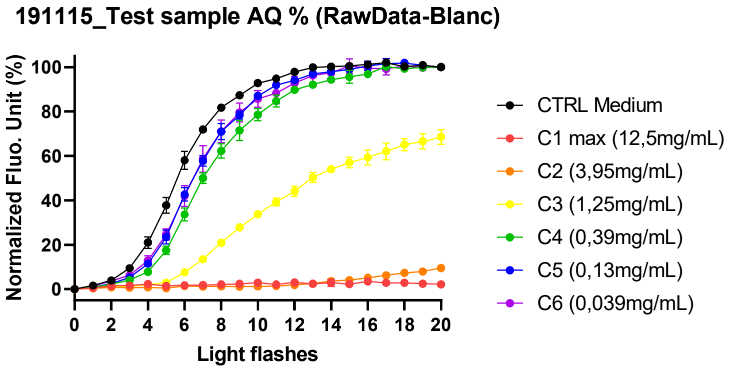

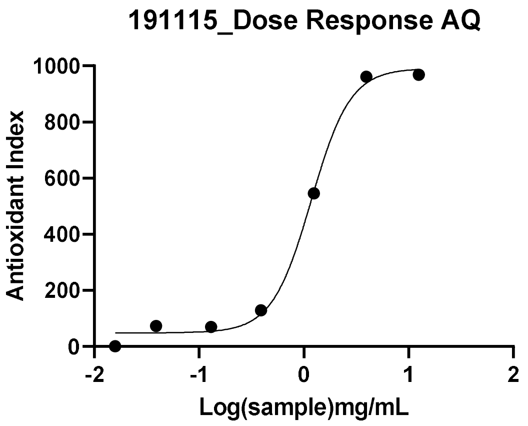

Sample AR

<u>Assay</u> : AOP 1
<u>Cellular model</u> : CaCo2

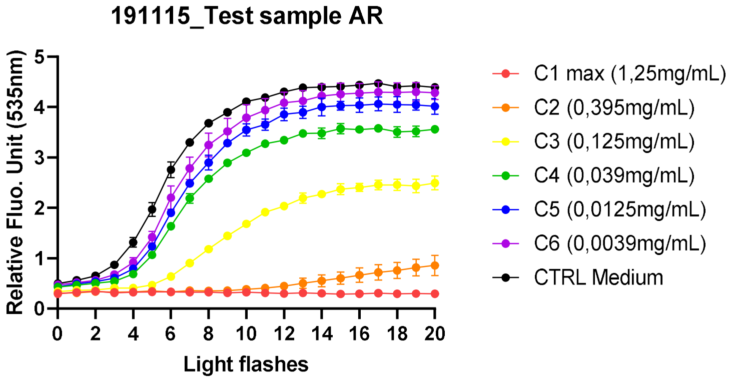

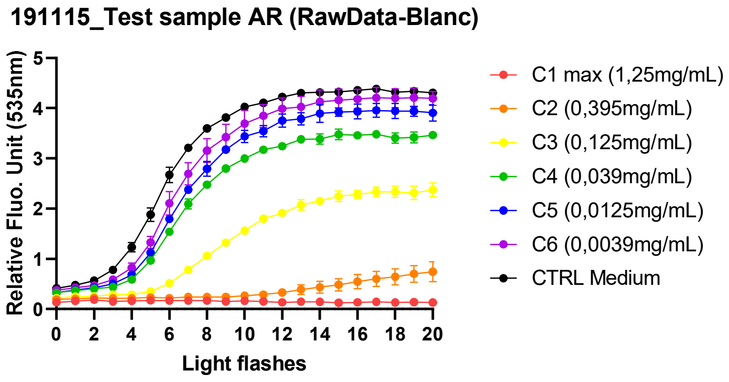

EC_{90} = 410.16 µg/ml
EC_{50} = 169.9 µg/ml
EC_{10} = 94.38 µg/ml
R^2 = 0.9962

Antioxidant index (normalized, max = 1000):
C1 : 988
C2 : 891
C3 : 243
C4 : 87
C5 : 71
C6 : 67

<u>Antioxidant index</u> :

$_0\int^{20}$ RFU_n control - $_0\int^{20}$ RFU_n sample

<u>Normalized antioxidant index</u> :

1000 – 1000 ($_0\int^{20}$ RFU_n sample /$_0\int^{20}$ RFU_n control)

<u>Graph legends</u> :

R^2 = Determination coefficient
EC_{50} = 50% efficacy concentration
Error bars correspond to +/- SD value of triplicates (3 wells)

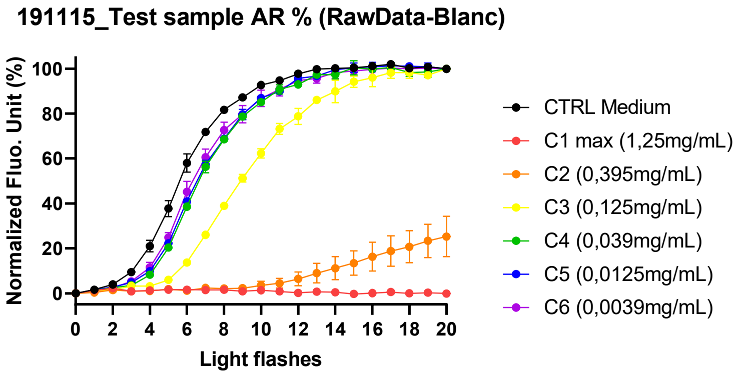

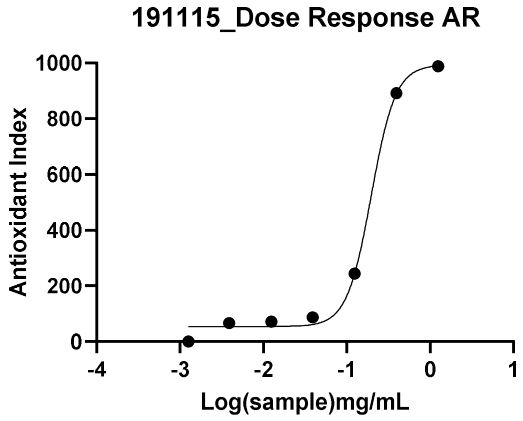

Sample AS

Assay : AOP 1
Cellular model : CaCo2

191115_Test sample AS

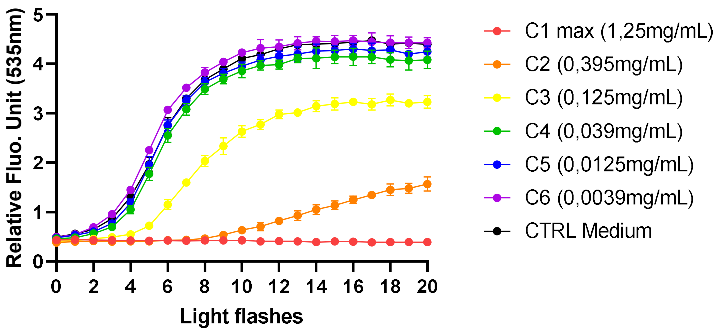

191115_Test sample AS (RawData-Blanc)

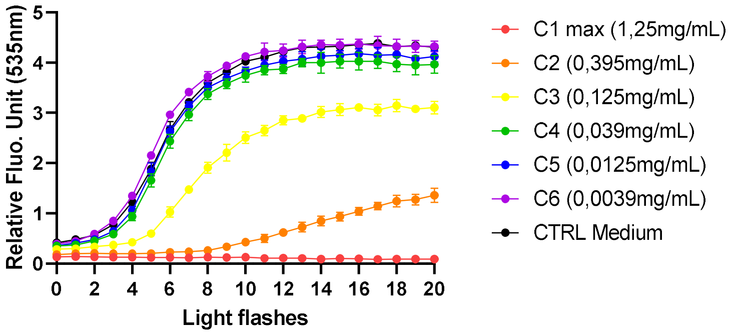

EC_{90} = 523.9 µg/ml
EC_{50} = 233.7 µg/ml
EC_{10} = 104.2 µg/ml
R^2 = 0.9996

Antioxidant index (normalized, max = 1000):
C1 : 991
C2 : 807
C3 : 148
C4 : 2
C5 : 1
C6 : -24

Antioxidant index :

$_0\int^{20} RFU_n$ control - $_0\int^{20} RFU_n$ sample

Normalized antioxidant index :

1000 – 1000 ($_0\int^{20} RFU_n$ sample / $_0\int^{20} RFU_n$ control)

Graph legends :

R^2 = Determination coefficient
EC_{50} = 50% efficacy concentration
Error bars correspond to +/- SD value of triplicates (3 wells)

191115_Test sample AS % (RawData-Blanc)

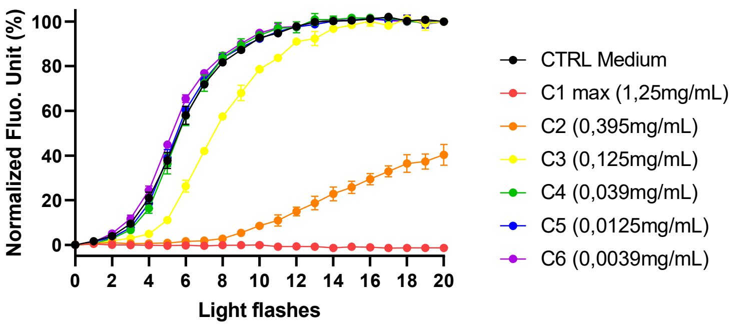

191115_Dose Response AS

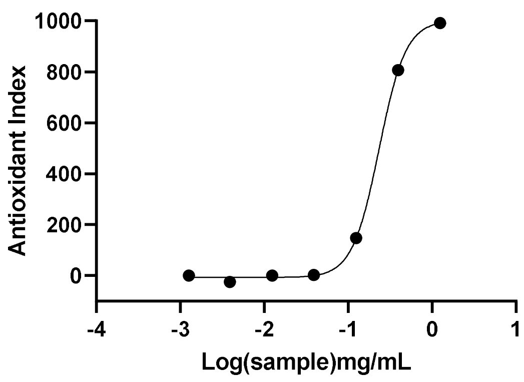

Sample AU

Assay : AOP 1
Cellular model : CaCo2

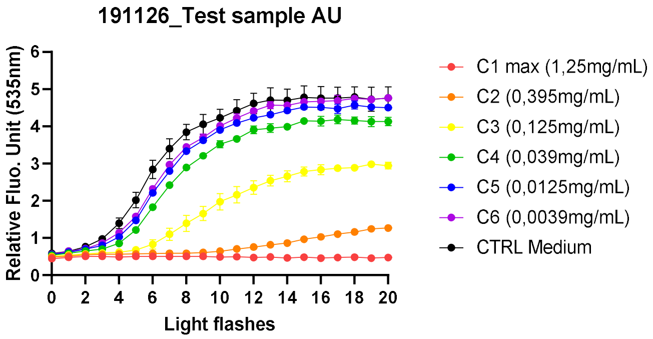

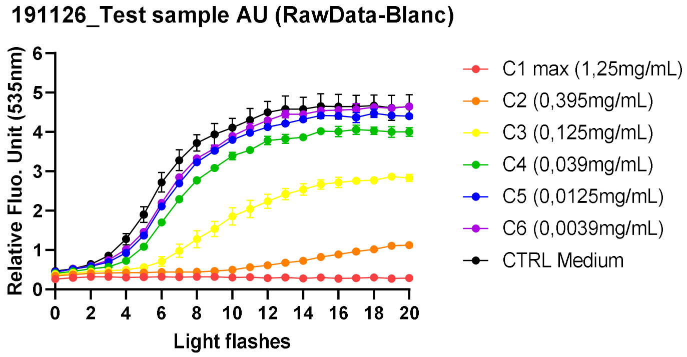

EC_{90} = 486.9 µg/ml
EC_{50} = 209.6 µg/ml
EC_{10} = 90.21 µg/ml
R^2 = 0.9975

Antioxidant index (normalized, max = 1000):
C1 : 975
C2 : 836
C3 : 234
C4 : 71
C5 : 49
C6 : 62

Antioxidant index :

${}_0\int^{20} RFU_n$ control - ${}_0\int^{20} RFU_n$ sample

Normalized antioxidant index :

1000 – 1000 (${}_0\int^{20} RFU_n$ sample /${}_0\int^{20} RFU_n$ control)

Graph legends :

R^2 = Determination coefficient
EC_{50} = 50% efficacy concentration
Error bars correspond to +/- SD value of triplicates (3 wells)

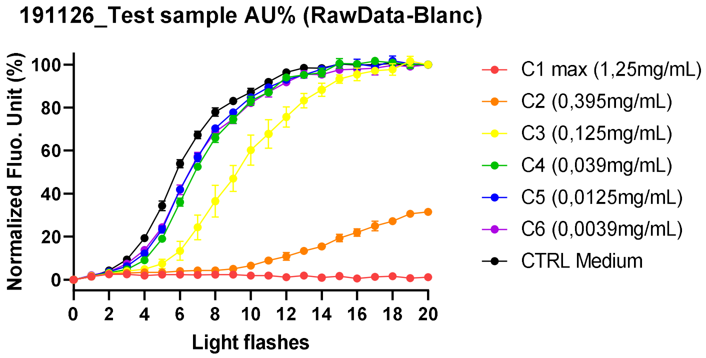

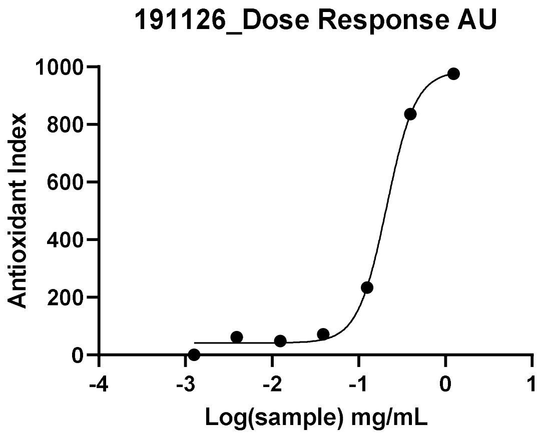

Sample AV

<u>Assay</u> : AOP 1
<u>Cellular model</u> : CaCo2

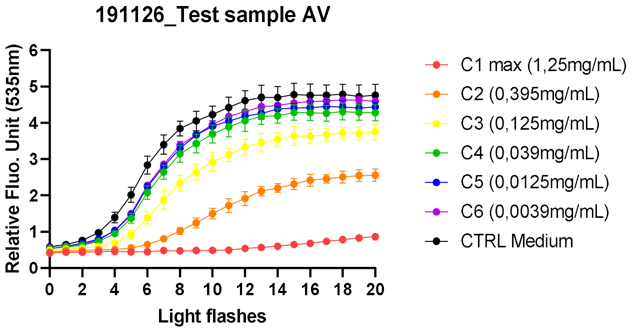

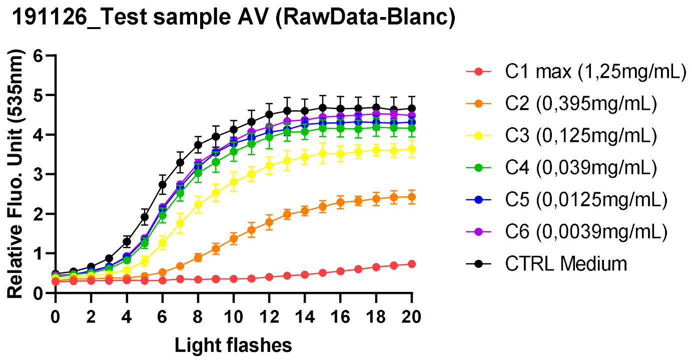

EC_{90} = 906.03 µg/ml
EC_{50} = 510.1 µg/ml
EC_{10} = 287.2 µg/ml
R^2 = 0.9906

Antioxidant index (normalized, max = 1000):
C1 : 903
C2 : 288
C3 : 137
C4 : 53
C5 : 43
C6 : 52

<u>Antioxidant index</u> :

$_0\int^{20}$ RFU_n control - $_0\int^{20}$ RFU_n sample

<u>Normalized antioxidant index</u> :

1000 – 1000 ($_0\int^{20}$ RFU_n sample / $_0\int^{20}$ RFU_n control)

<u>Graph legends</u> :

R^2 = Determination coefficient
EC_{50} = 50% efficacy concentration
Error bars correspond to +/- SD value of triplicates (3 wells)

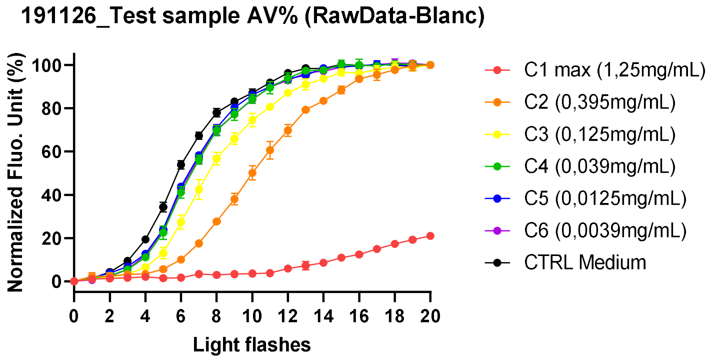

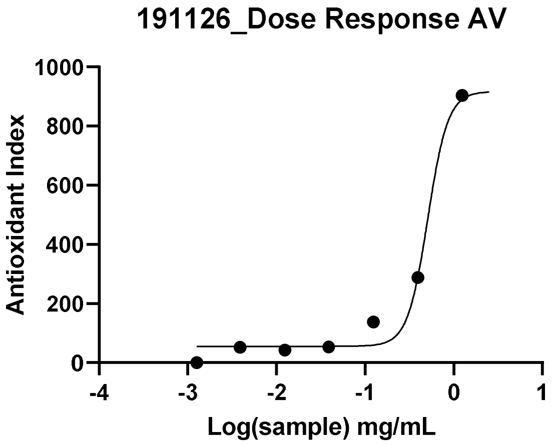

Sample AW

Assay : AOP 1
Cellular model : CaCo2

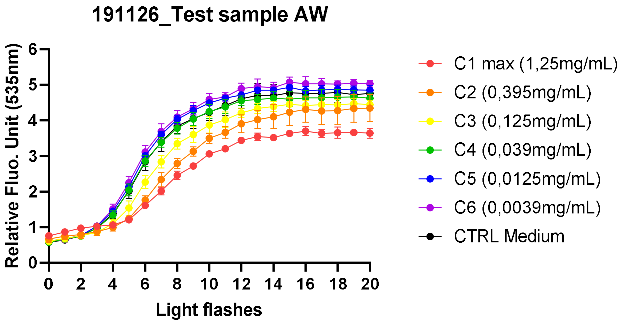

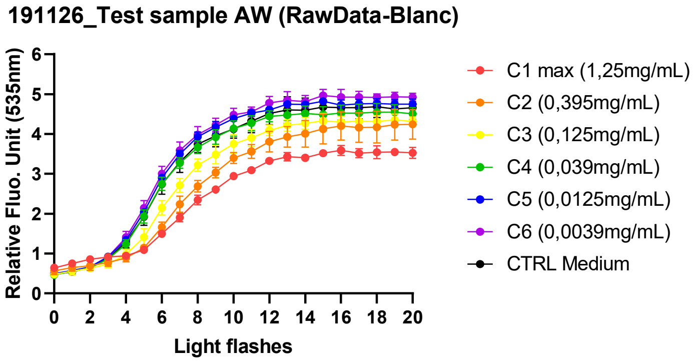

EC_{90} = ND
EC_{50} = ND
EC_{10} = ND
R^2 = ND

Antioxidant index (normalized, max = 1000):
C1 : 90
C2 : 119
C3 : 41
C4 : -17
C5 : -20
C6 : -11

Antioxidant index :

$_0\int^{20} RFU_n$ control - $_0\int^{20} RFU_n$ sample

Normalized antioxidant index :

$1000 - 1000\ ({}_0\int^{20} RFU_n \text{ sample} / {}_0\int^{20} RFU_n \text{ control})$

Graph legends :

R^2 = Determination coefficient
EC_{50} = 50% efficacy concentration
Error bars correspond to +/- SD value of triplicates (3 wells)

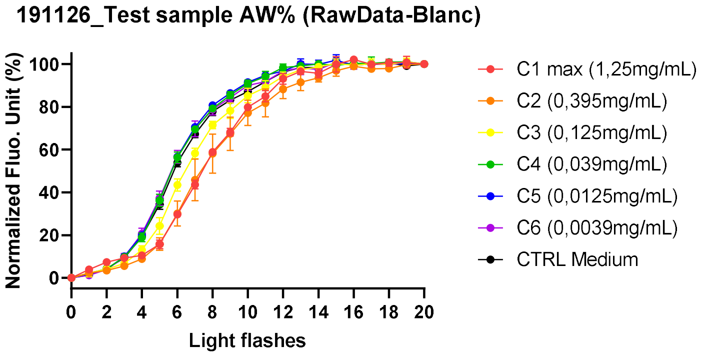

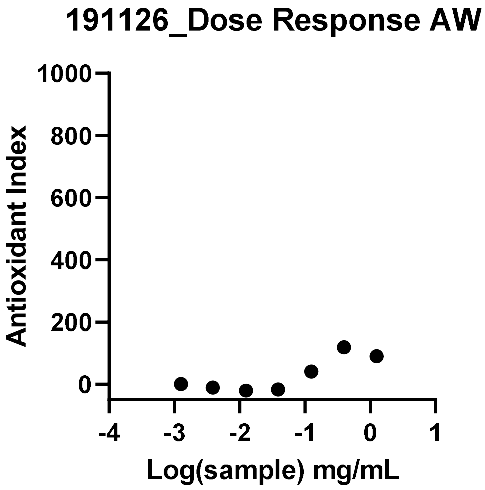

Sample AX

Assay : AOP 1
Cellular model : CaCo2

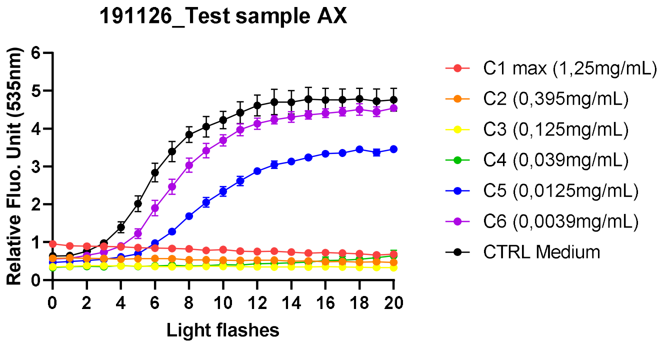

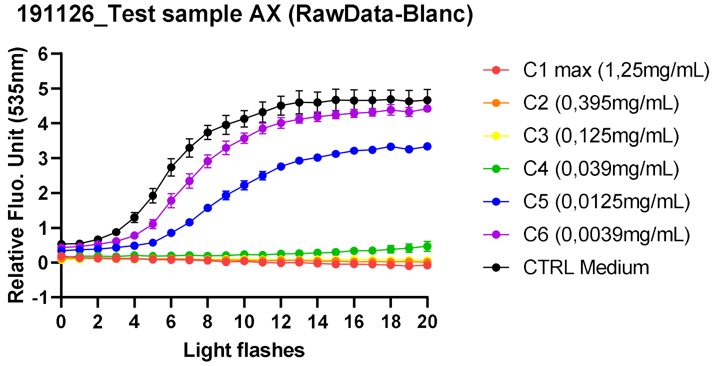

EC_{90} = 32.19 µg/ml
EC_{50} = 18.58 µg/ml
EC_{10} = 10.73 µg/ml
R^2 = 0.9945

Antioxidant index (normalized, max = 1000):
C3 : 995
C4 : 952
C5 : 209
C6 : 102

Antioxidant index :

${}_0\int^{20}$ RFU_n control - ${}_0\int^{20}$ RFU_n sample

Normalized antioxidant index :

1000 – 1000 (${}_0\int^{20}$ RFU_n sample /${}_0\int^{20}$ RFU_n control)

Graph legends :

R^2 = Determination coefficient
EC_{50} = 50% efficacy concentration
Error bars correspond to +/- SD value of triplicates (3 wells)

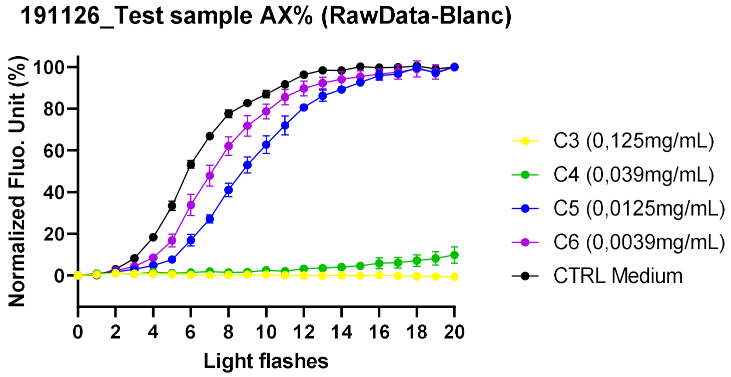

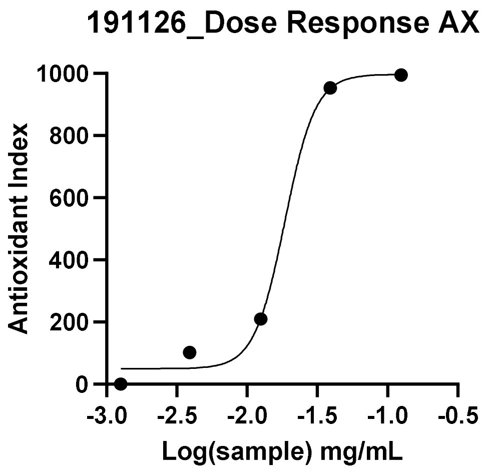

Sample AY

Assay : AOP 1
Cellular model : CaCo2

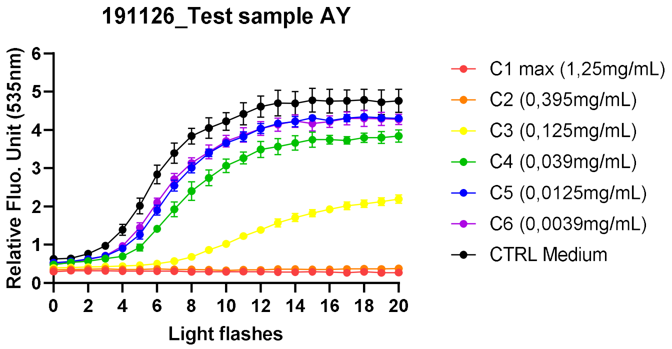

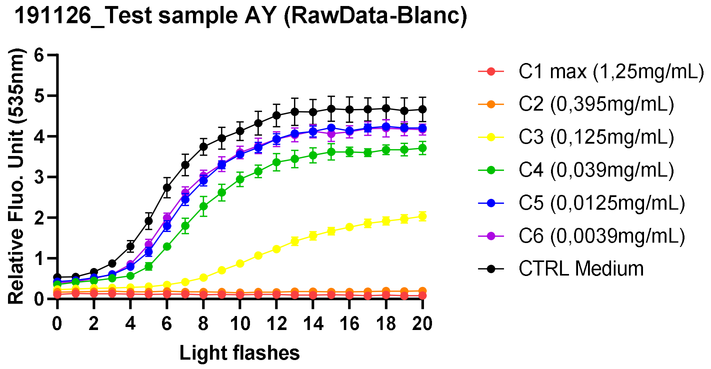

EC_{90} = 224.1 µg/ml
EC_{50} = 95.43 µg/ml
EC_{10} = 40.65 µg/ml
R^2 = 0.9982

Antioxidant index (normalized, max = 1000):
C1 : 995
C2 : 996
C3 : 682
C4 : 123
C5 : 66
C6 : 44

Antioxidant index :

$_0\int^{20}$ RFU$_n$ control - $_0\int^{20}$ RFU$_n$ sample

Normalized antioxidant index :

1000 – 1000 ($_0\int^{20}$ RFU$_n$ sample /$_0\int^{20}$ RFU$_n$ control)

Graph legends :

R^2 = Determination coefficient
EC_{50} = 50% efficacy concentration
Error bars correspond to +/- SD value of triplicates (3 wells)

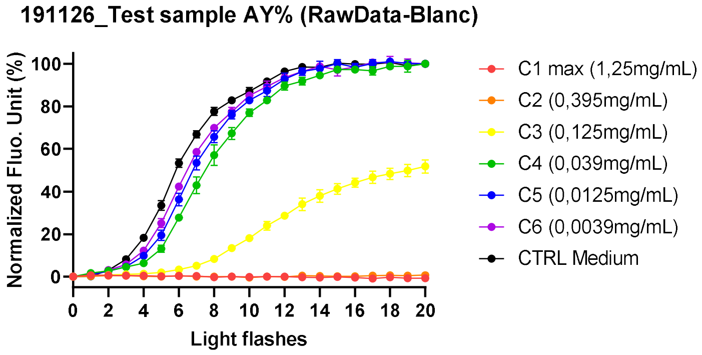

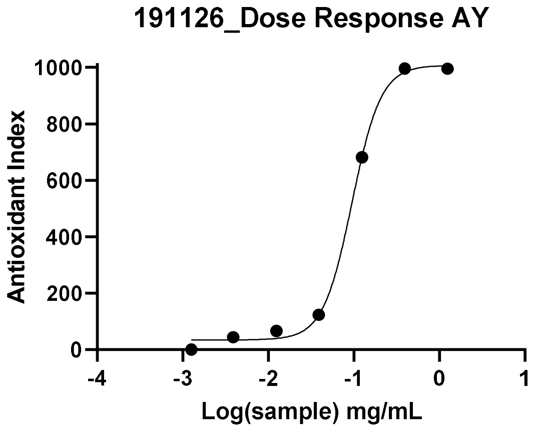

Sample AZ

Assay : AOP 1
Cellular model : CaCo2

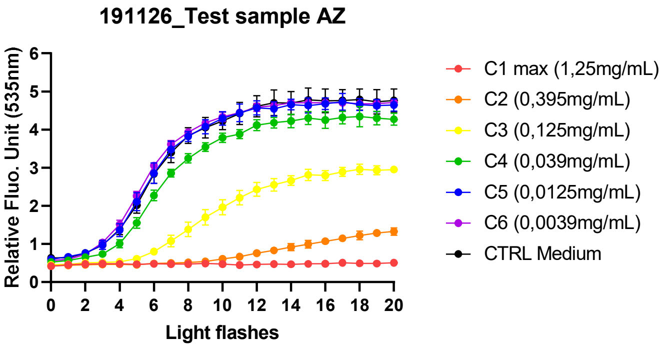

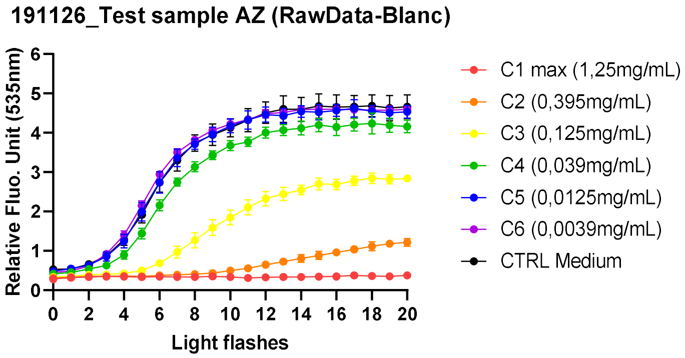

EC_{90} = 503 µg/ml
EC_{50} = 200.5 µg/ml
EC_{10} = 79.97 µg/ml
R^2 = 0.9992

Antioxidant index (normalized, max = 1000):
C1 : 966
C2 : 818
C3 : 225
C4 : 21
C5 : -23
C6 : -31

Antioxidant index :

$_0\int^{20}$ RFU$_n$ control - $_0\int^{20}$ RFU$_n$ sample

Normalized antioxidant index :

1000 – 1000 ($_0\int^{20}$ RFU$_n$ sample / $_0\int^{20}$ RFU$_n$ control)

Graph legends :

R^2 = Determination coefficient
EC_{50} = 50% efficacy concentration
Error bars correspond to +/- SD value of triplicates (3 wells)

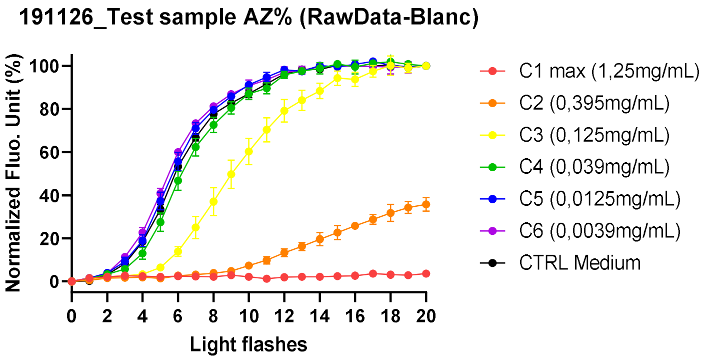

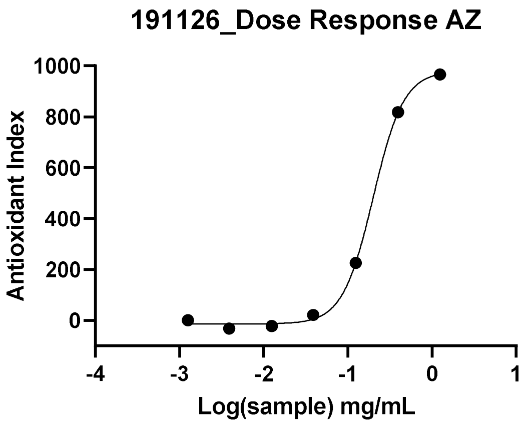

Sample BA

Assay : AOP 1
Cellular model : CaCo2

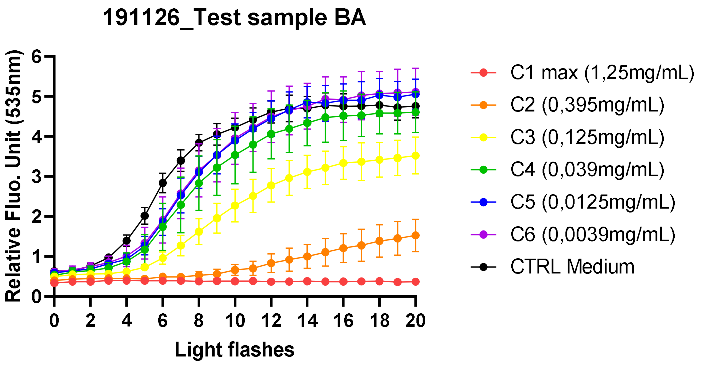

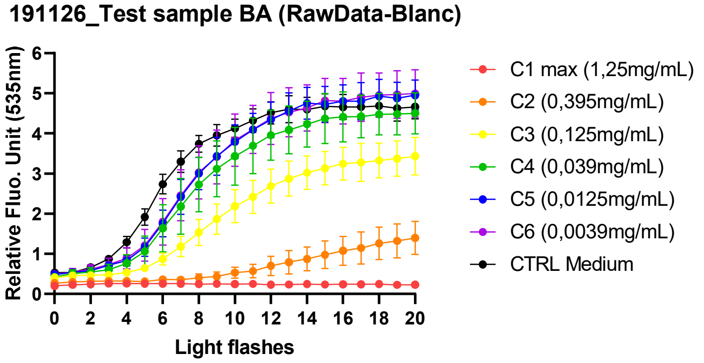

EC_{90} = 531.2 µg/ml
EC_{50} = 229.6 µg/ml
EC_{10} = 99.2 µg/ml
R^2 = 0.9861

Antioxidant index (normalized, max = 1000):
C1 : 981
C2 : 820
C3 : 243
C4 : 130
C5 : 131
C6 : 133

Antioxidant index :

$_0\int^{20}$ RFU_n control - $_0\int^{20}$ RFU_n sample

Normalized antioxidant index :

1000 – 1000 ($_0\int^{20}$ RFU_n sample / $_0\int^{20}$ RFU_n control)

Graph legends :

R^2 = Determination coefficient
EC_{50} = 50% efficacy concentration
Error bars correspond to +/- SD value of triplicates (3 wells)

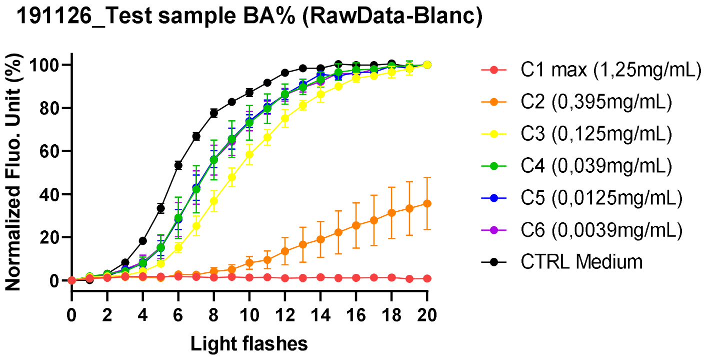

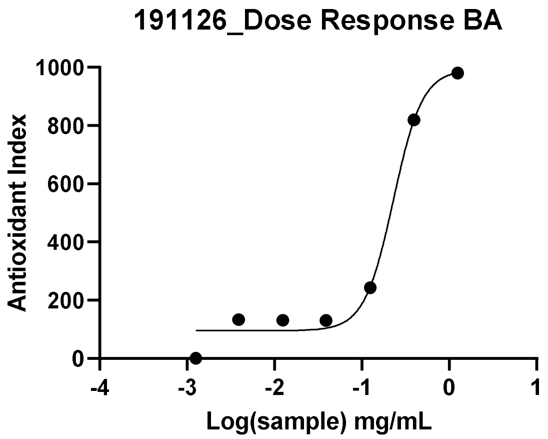

Sample BB

Assay : AOP 1
Cellular model : CaCo2

191126_Test sample BB

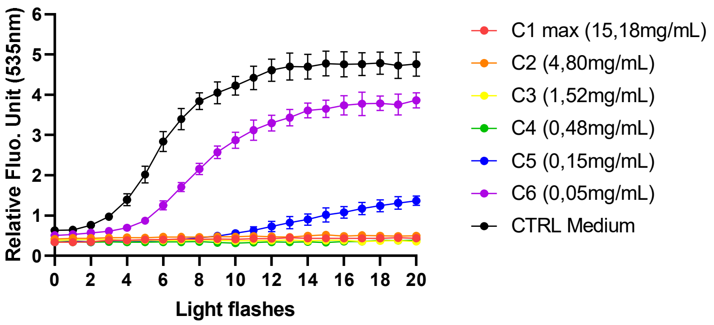

191126_Test sample BB (RawData-Blanc)

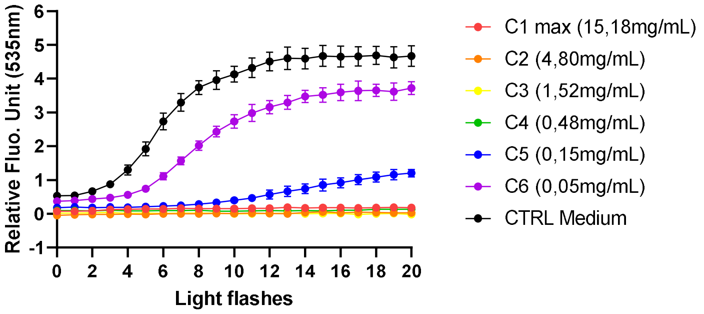

EC_{90} = 169.1 µg/ml
EC_{50} = 80.65 µg/ml
EC_{10} = 38.45 µg/ml
R^2 = 0.9994

Antioxidant index (normalized, max = 1000):
C1 : 970
C2 : 979
C3 : 996
C4 : 996
C5 : 854
C6 : 171

Antioxidant index :

$_0\int^{20}$ RFU$_n$ control - $_0\int^{20}$ RFU$_n$ sample

Normalized antioxidant index :

1000 – 1000 ($_0\int^{20}$ RFU$_n$ sample /$_0\int^{20}$ RFU$_n$ control)

Graph legends :

R^2 = Determination coefficient
EC_{50} = 50% efficacy concentration
Error bars correspond to +/- SD value of triplicates (3 wells)

191126_Test sample BB% (RawData-Blanc)

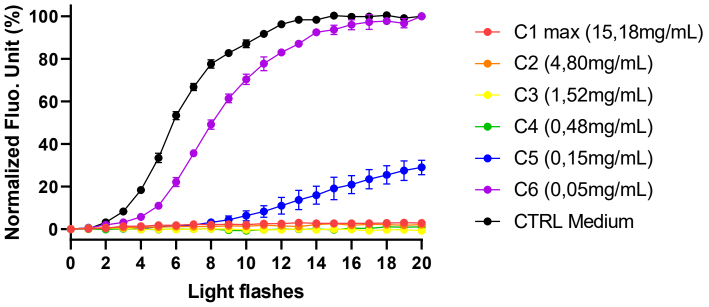

191126_Dose Response BB

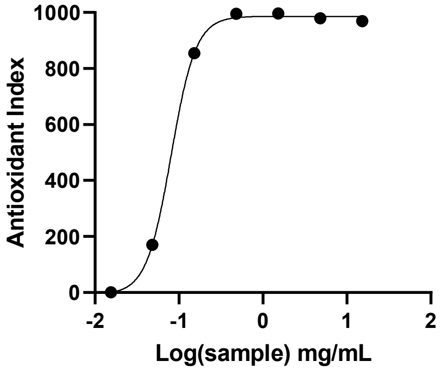

Sample BC

Assay : AOP 1
Cellular model : CaCo2

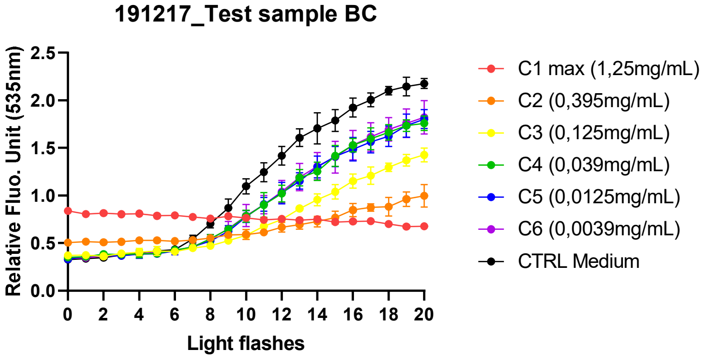

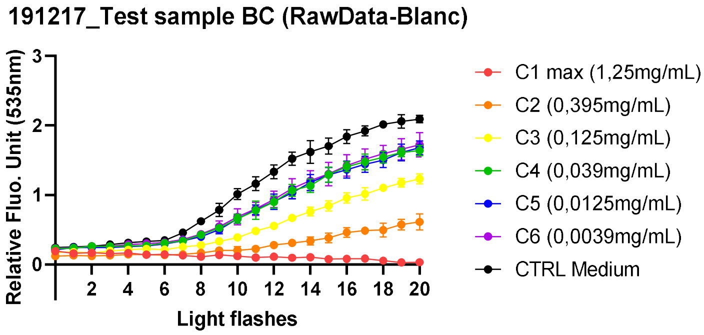

EC_{90} = 380.5 µg/ml
EC_{50} = 143.6 µg/ml
EC_{10} = 54.21µg/ml
R^2 = 0.9951

Antioxidant index (normalized, max = 1000):
C1 : 858
C2 : 740
C3 : 366
C4 : -13
C6 : 11

Antioxidant index :

$_0\int^{20} RFU_n$ control - $_0\int^{20} RFU_n$ sample

Normalized antioxidant index :

1000 – 1000 ($_0\int^{20} RFU_n$ sample /$_0\int^{20} RFU_n$ control)

Graph legends :

R^2 = Determination coefficient
EC_{50} = 50% efficacy concentration
Error bars correspond to +/- SD value of triplicates (3 wells)

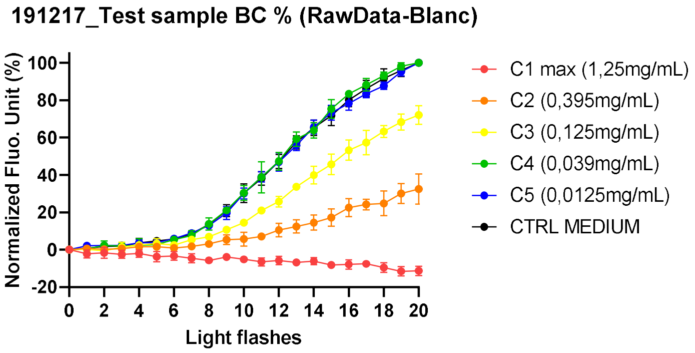

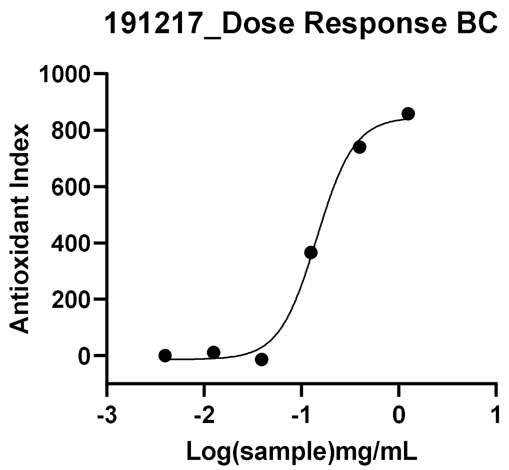

Sample BD

Assay : AOP 1
Cellular model : CaCo2

191217_Test sample BD

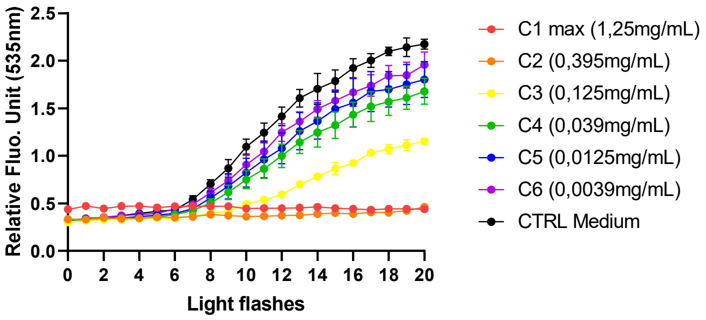

191217_Test sample BD (RawData-Blanc)

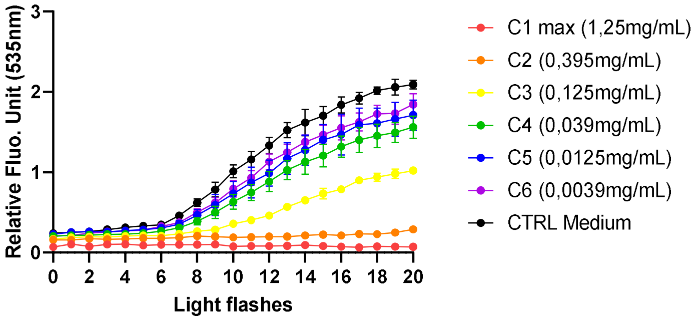

EC_{90} = 364 µg/ml
EC_{50} = 201.6 µg/ml
EC_{10} = 111.7 µg/ml
R^2 = 0.9985

Antioxidant index (normalized, max = 1000):
C1 : 956
C2 : 888
C3 : 156
C4 : 55
C5 : 13

Antioxidant index :

$_0\int^{20}$ RFU_n control - $_0\int^{20}$ RFU_n sample

Normalized antioxidant index :

1000 – 1000 ($_0\int^{20}$ RFU_n sample /$_0\int^{20}$ RFU_n control)

Graph legends :

R^2 = Determination coefficient
EC_{50} = 50% efficacy concentration
Error bars correspond to +/- SD value of triplicates (3 wells)

191217_Test sample BD % (RawData-Blanc)

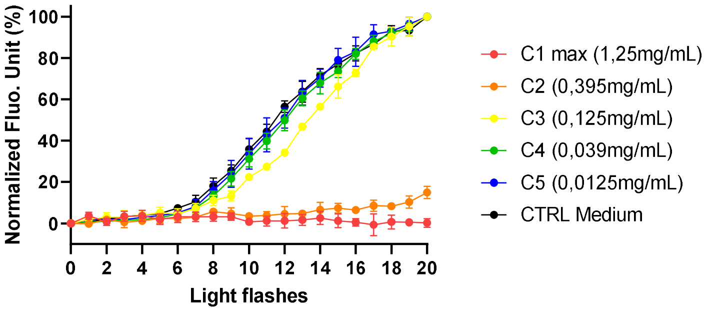

191217_Dose Response BD

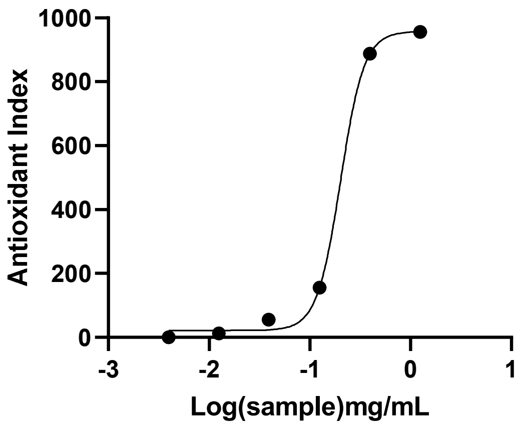

Sample BE

Assay : AOP 1
Cellular model : CaCo2

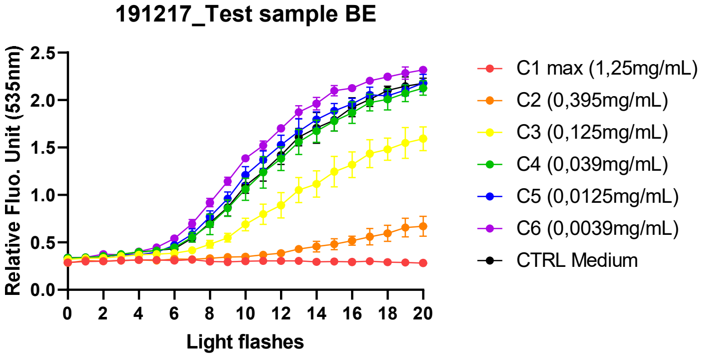

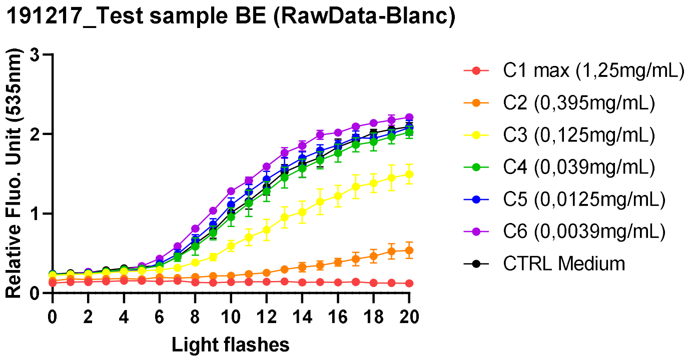

EC_{90} = 539.6 µg/ml
EC_{50} = 226.1 µg/ml
EC_{10} = 94.75 µg/ml
R^2 = 0.9948

Antioxidant index (normalized, max = 1000):
C1 : 979
C2 : 812
C3 : 215
C4 : 105
C5 : 61

Antioxidant index :

$_0\int^{20}$ RFU_n control - $_0\int^{20}$ RFU_n sample

Normalized antioxidant index :

1000 – 1000 ($_0\int^{20}$ RFU_n sample /$_0\int^{20}$ RFU_n control)

Graph legends :

R^2 = Determination coefficient
EC_{50} = 50% efficacy concentration
Error bars correspond to +/- SD value of triplicates (3 wells)

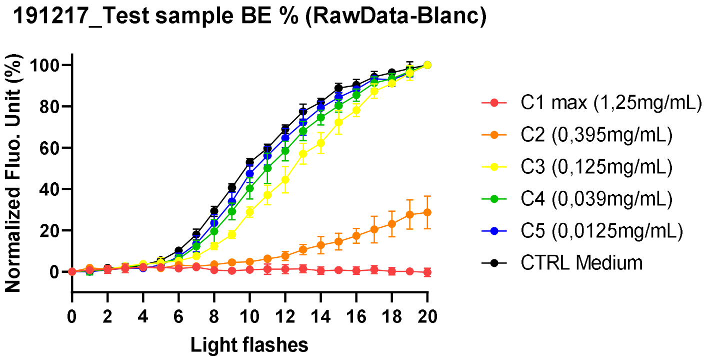

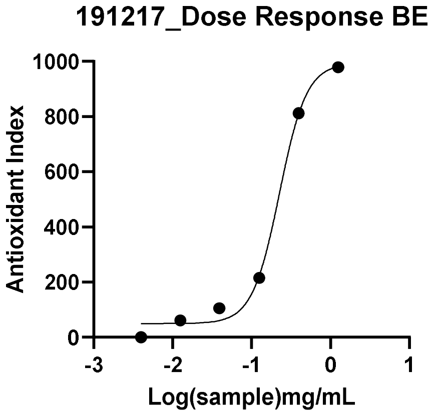

Sample BF

Assay : AOP 1
Cellular model : CaCo2

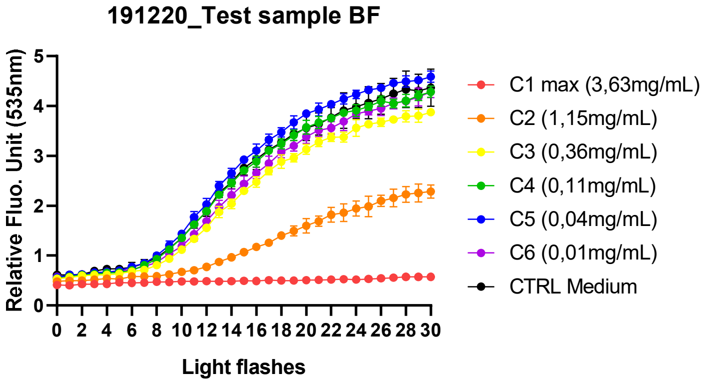

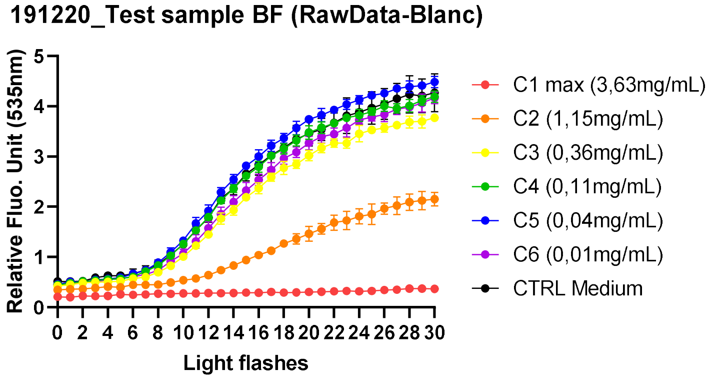

EC_{90} = 2009 µg/ml
EC_{50} = 1421 µg/ml
EC_{10} = 1005 µg/ml
R^2 = 0.9965

Antioxidant index (normalized, max = 1000):
C1 : 888
C2 : 187
C3 : 22
C4 : -19
C5 : -28
C6 : 50

Antioxidant index :

$_0\int^{30}$ RFU_n control - $_0\int^{30}$ RFU_n sample

Normalized antioxidant index :

1000 – 1000 ($_0\int^{30}$ RFU_n sample / $_0\int^{30}$ RFU_n control)

Graph legends :

R^2 = Determination coefficient
EC_{50} = 50% efficacy concentration
Error bars correspond to +/- SD value of triplicates (3 wells)

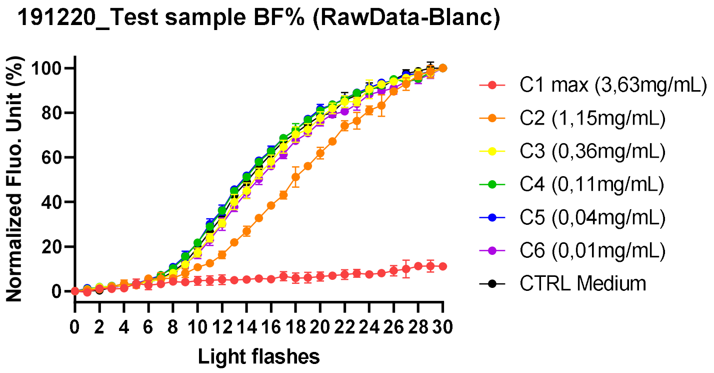

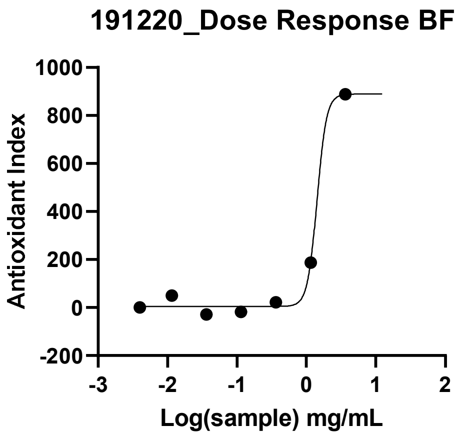

Supplementary data - Figure S2 - AOP 1 on HepG2 cells (pre-apical and basolateral compartments)

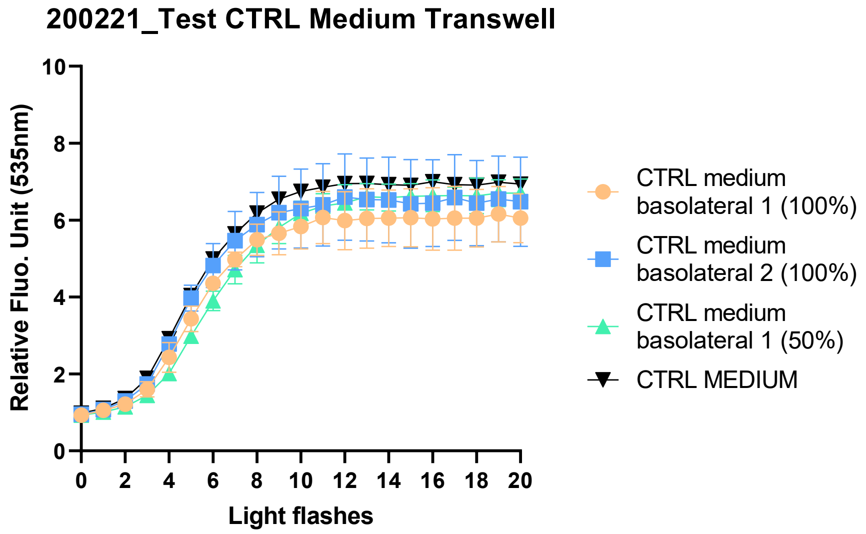

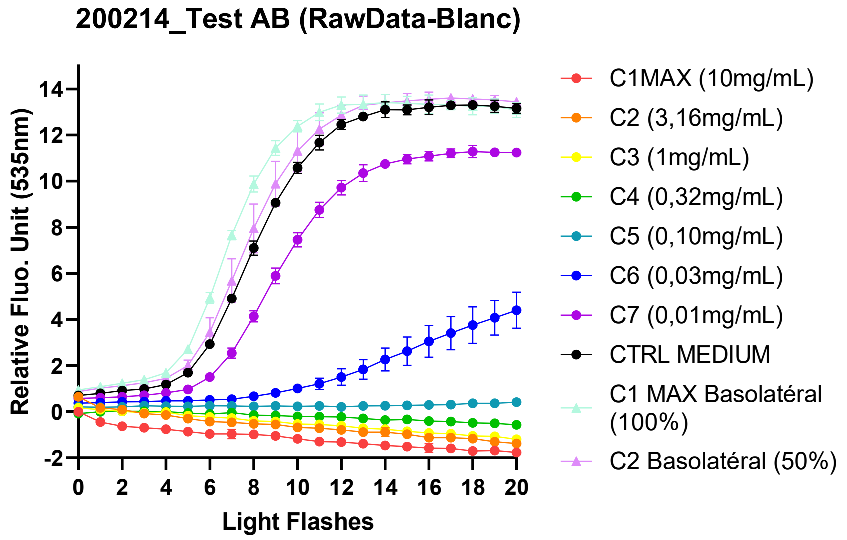

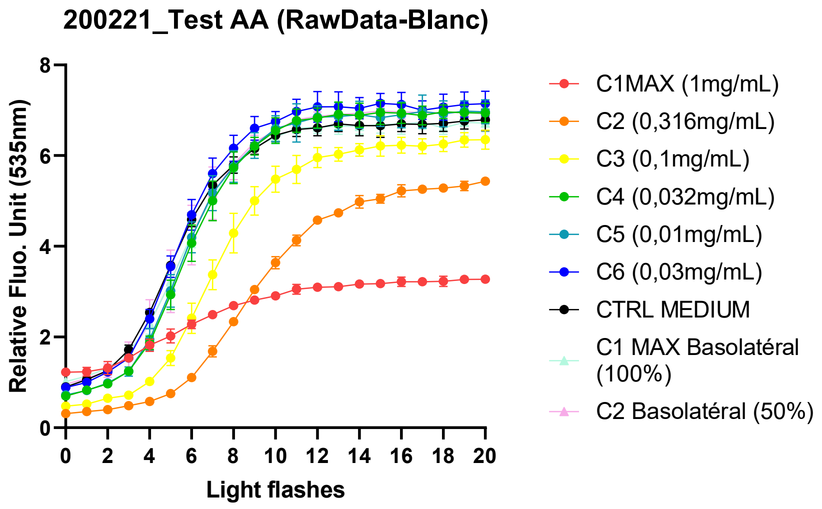

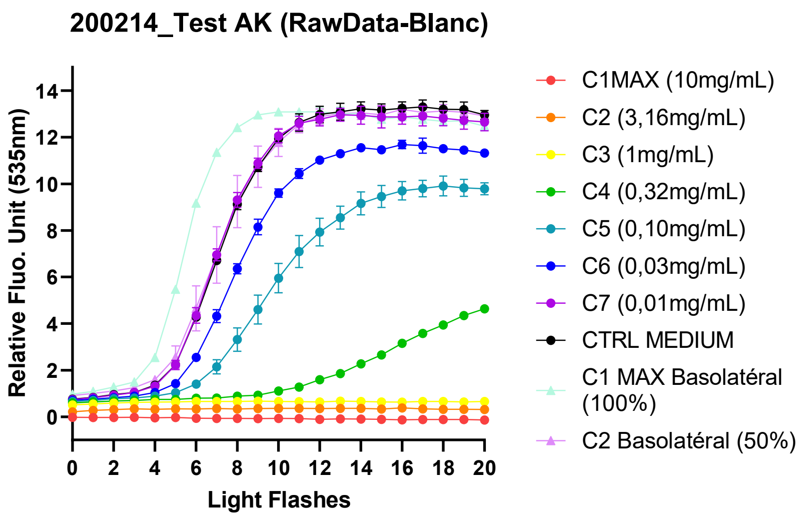
200214_Test AK (RawData-Blanc)
Relative Fluo. Unit (535nm)
14
12
10
8
6
4
2
0
0 2 4 6 8 10 12 14 16 18 20
Light Flashes
C1MAX (10mg/mL)
C2 (3,16mg/mL)
C3 (1mg/mL)
C4 (0,32mg/mL)
C5 (0,10mg/mL)
C6 (0,03mg/mL)
C7 (0,01mg/mL)
CTRL MEDIUM
C1 MAX Basolatéral (100%)
C2 Basolatéral (50%)

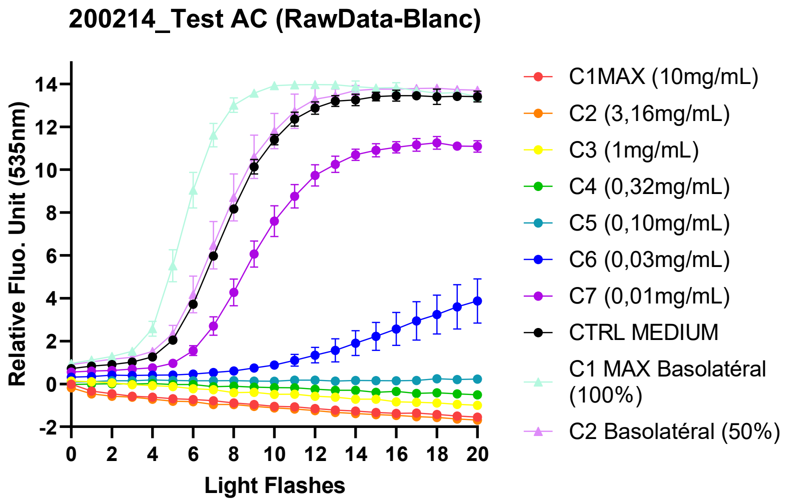
200214_Test AC (RawData-Blanc)
Relative Fluo. Unit (535nm)
14
12
10
8
6
4
2
0
-2
0 2 4 6 8 10 12 14 16 18 20
Light Flashes
C1MAX (10mg/mL)
C2 (3,16mg/mL)
C3 (1mg/mL)
C4 (0,32mg/mL)
C5 (0,10mg/mL)
C6 (0,03mg/mL)
C7 (0,01mg/mL)
CTRL MEDIUM
C1 MAX Basolatéral (100%)
C2 Basolatéral (50%)

200214_Test AL (RawData-Blanc)

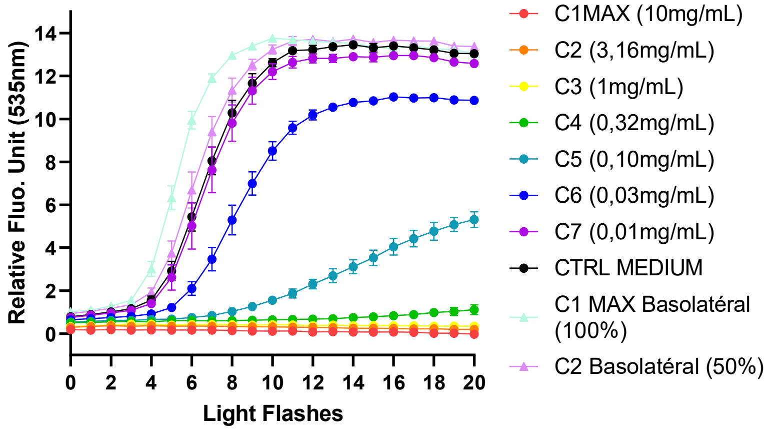

200214_Test AM (RawData-Blanc)

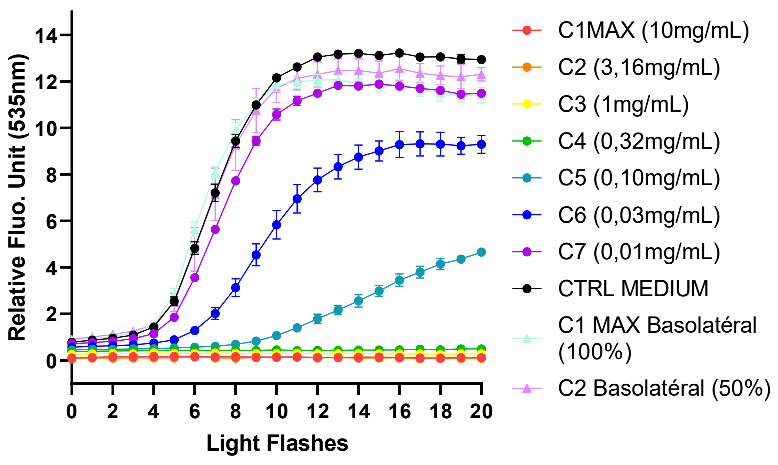

200214_Test AN (RawData-Blanc)

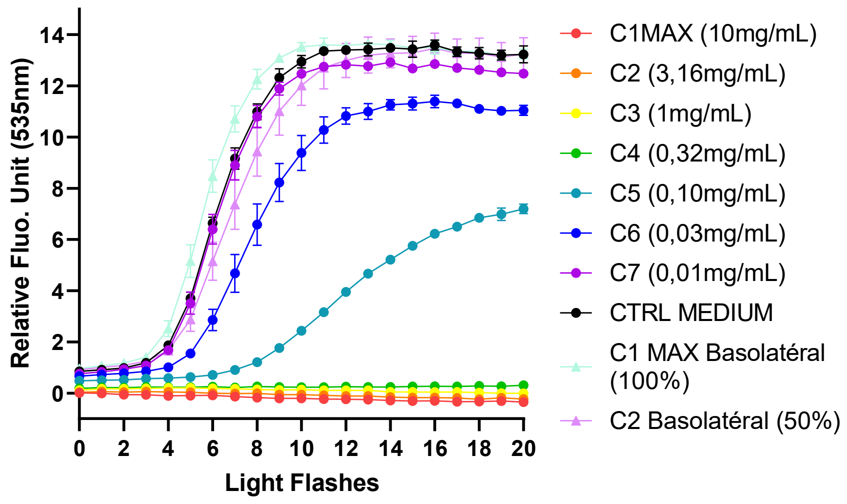

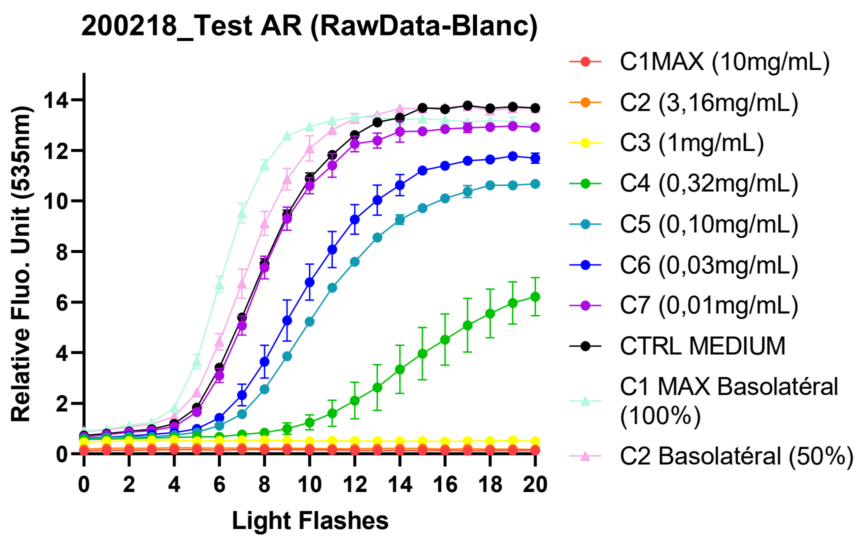
200218_Test AR (RawData-Blanc)
Relative Fluo. Unit (535nm)
Light Flashes
C1MAX (10mg/mL)
C2 (3,16mg/mL)
C3 (1mg/mL)
C4 (0,32mg/mL)
C5 (0,10mg/mL)
C6 (0,03mg/mL)
C7 (0,01mg/mL)
CTRL MEDIUM
C1 MAX Basolatéral (100%)
C2 Basolatéral (50%)

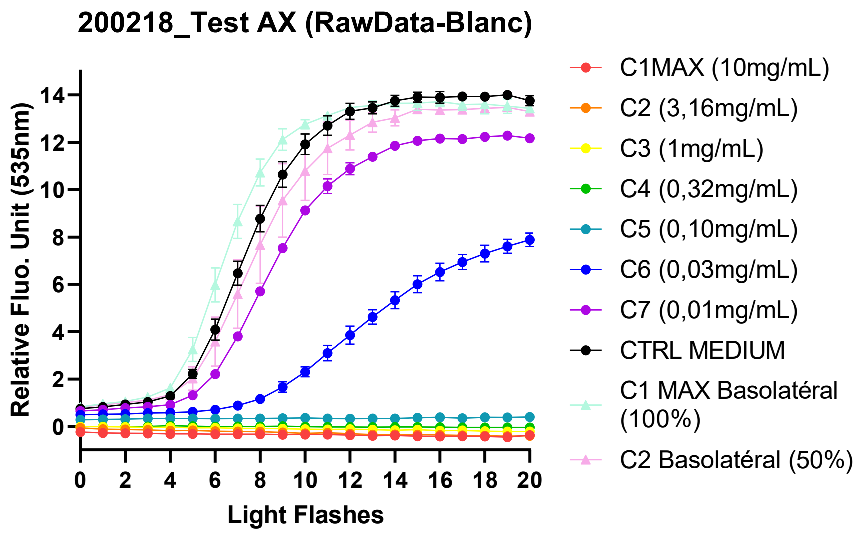
200218_Test AX (RawData-Blanc)
Relative Fluo. Unit (535nm)
Light Flashes
C1MAX (10mg/mL)
C2 (3,16mg/mL)
C3 (1mg/mL)
C4 (0,32mg/mL)
C5 (0,10mg/mL)
C6 (0,03mg/mL)
C7 (0,01mg/mL)
CTRL MEDIUM
C1 MAX Basolatéral (100%)
C2 Basolatéral (50%)

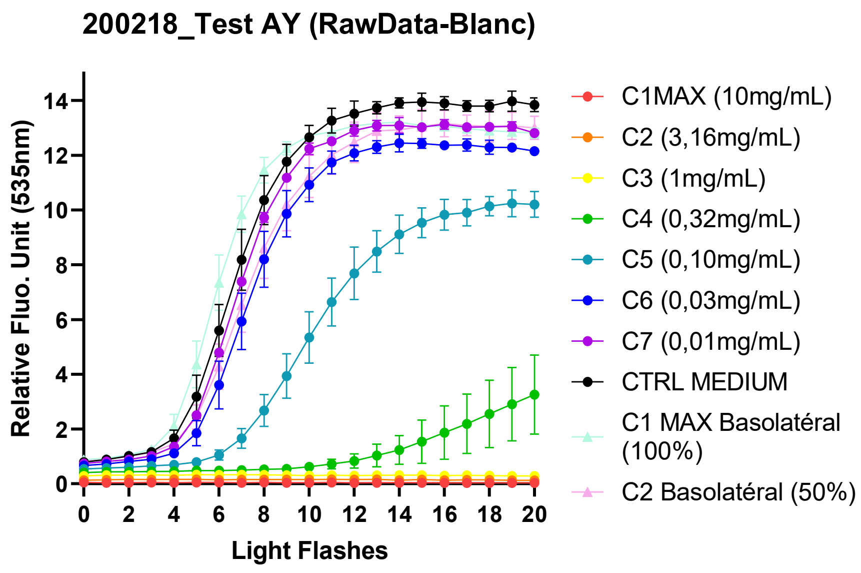
200218_Test AY (RawData-Blanc)
Relative Fluo. Unit (535nm)
Light Flashes
C1MAX (10mg/mL)
C2 (3,16mg/mL)
C3 (1mg/mL)
C4 (0,32mg/mL)
C5 (0,10mg/mL)
C6 (0,03mg/mL)
C7 (0,01mg/mL)
CTRL MEDIUM
C1 MAX Basolatéral (100%)
C2 Basolatéral (50%)

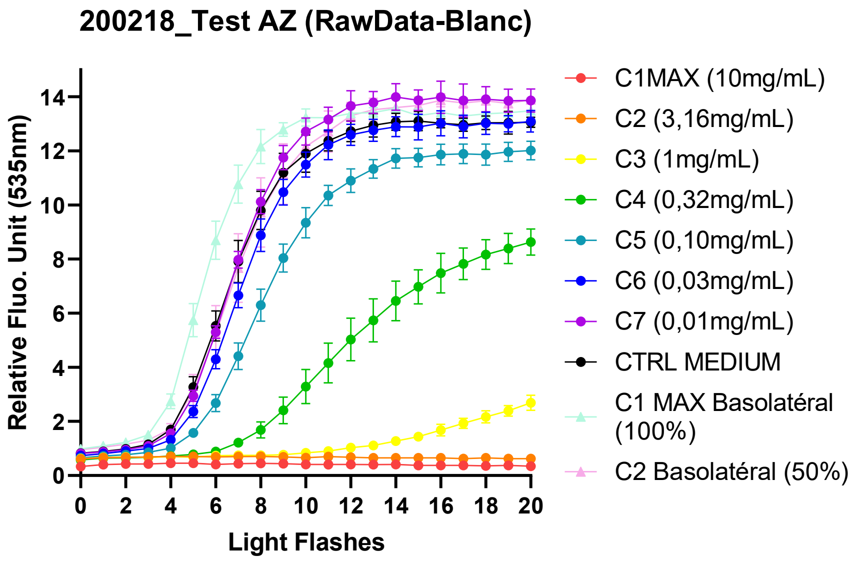
200218_Test AZ (RawData-Blanc)
Relative Fluo. Unit (535nm)
Light Flashes
0
2
4
6
8
10
12
14
16
18
20
C1MAX (10mg/mL)
C2 (3,16mg/mL)
C3 (1mg/mL)
C4 (0,32mg/mL)
C5 (0,10mg/mL)
C6 (0,03mg/mL)
C7 (0,01mg/mL)
CTRL MEDIUM
C1 MAX Basolatéral (100%)
C2 Basolatéral (50%)

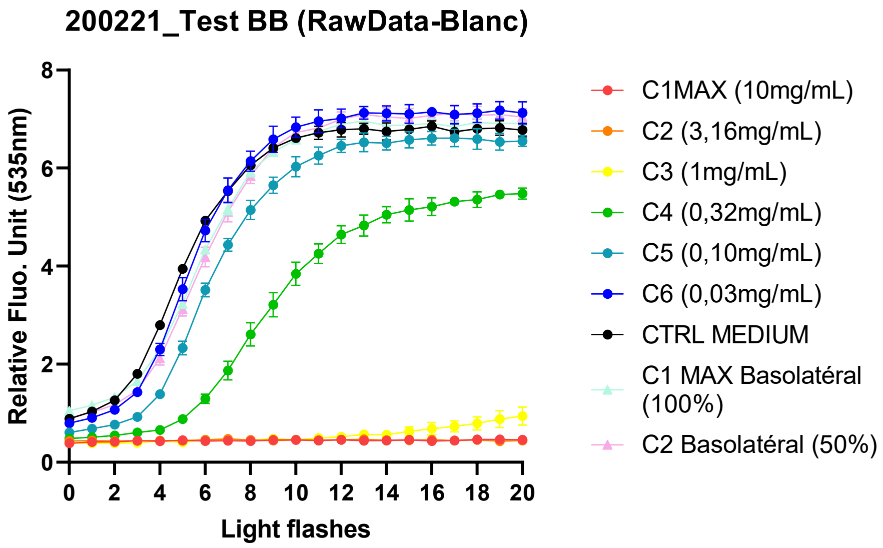
200221_Test BB (RawData-Blanc)
Relative Fluo. Unit (535nm)
Light flashes
0
2
4
6
8
10
12
14
16
18
20
C1MAX (10mg/mL)
C2 (3,16mg/mL)
C3 (1mg/mL)
C4 (0,32mg/mL)
C5 (0,10mg/mL)
C6 (0,03mg/mL)
CTRL MEDIUM
C1 MAX Basolatéral (100%)
C2 Basolatéral (50%)
